# Metabolic ripple effects – deciphering how lipid metabolism in cancer interfaces with the tumor microenvironment

**DOI:** 10.1242/dmm.050814

**Published:** 2024-09-16

**Authors:** Patrick B. Jonker, Alexander Muir

**Affiliations:** Ben May Department for Cancer Research, University of Chicago, Chicago, IL 60637, USA

**Keywords:** Acidosis, Diet, Hypoxia, Lipid metabolism, Nutrient deprivation, Tumor microenvironment

## Abstract

Cancer cells require a constant supply of lipids. Lipids are a diverse class of hydrophobic molecules that are essential for cellular homeostasis, growth and survival, and energy production. How tumors acquire lipids is under intensive investigation, as these mechanisms could provide attractive therapeutic targets for cancer. Cellular lipid metabolism is tightly regulated and responsive to environmental stimuli. Thus, lipid metabolism in cancer is heavily influenced by the tumor microenvironment. In this Review, we outline the mechanisms by which the tumor microenvironment determines the metabolic pathways used by tumors to acquire lipids. We also discuss emerging literature that reveals that lipid availability in the tumor microenvironment influences many metabolic pathways in cancers, including those not traditionally associated with lipid biology. Thus, metabolic changes instigated by the tumor microenvironment have ‘ripple’ effects throughout the densely interconnected metabolic network of cancer cells. Given the interconnectedness of tumor metabolism, we also discuss new tools and approaches to identify the lipid metabolic requirements of cancer cells in the tumor microenvironment and characterize how these requirements influence other aspects of tumor metabolism.

## Teaser

**Summary:** This Review discusses how the tumor microenvironment regulates lipid metabolism in cancers and how lipid metabolism in cancers reciprocally affects tumor metabolism and biology.

## Introduction

Altered regulation of cellular metabolism is considered a hallmark of cancer (Hanahan, 2022). Rapidly proliferating, transformed cancer cells require metabolic adaptations to support their enhanced energetic and biosynthetic demands (Hanahan, 2022; Vander Heiden and DeBerardinis, 2017). Because metabolic changes are necessary for tumor growth, ongoing efforts in cancer research seek to identify key nodes in these metabolic adaptations that might provide potential, novel targets for anti-cancer therapies (Stine et al., 2022).

However, these efforts have been hampered by various challenges (Fendt et al., 2020; Martínez-Reyes and Chandel, 2021; Stine et al., 2022). In particular, cell-extrinsic features in the tumor microenvironment (TME), such as limited oxygen, pathophysiological nutrient availability, acidity, the presence of stromal cells and physical stressors, influence the metabolism and targetable metabolic liabilities of cancer cells (Faubert et al., 2020; Lau and Vander Heiden, 2020; Lyssiotis and Kimmelman, 2017; Muir et al., 2018). As cancer cell metabolism is influenced by features outside of the cell, the metabolic properties and liabilities of cancer cells defined in one experimental model system might not be relevant in a clinical setting, in which cancer cells face different external stimuli (Elia and Haigis, 2021; Lau and Vander Heiden, 2020; Lim et al., 2020; Lyssiotis and Kimmelman, 2017; Muir and Vander Heiden, 2018). Thus, efforts to identify clinically relevant metabolic vulnerabilities of cancer cells must effectively model diverse components of the TME.

Lipid metabolism is required for cellular biomass and energy production. It is well established that oncogenic mutations and the signaling pathways downstream of these mutated oncogenes regulate lipid metabolic pathways in cancer cells to enable tumor growth and proliferation. For comprehensive reviews on how oncogenic mutations in cancer cells rewire cellular lipid metabolism, we refer readers to these excellent reviews: Dang and Semenza (1999); Iurlaro et al. (2014); Nagarajan et al. (2016); Snaebjornsson et al. (2020); and Vogel et al. (2024). However, recent research suggests that lipid metabolism in cancer cells also responds to factors in the TME. In the first part of this Review, we discuss this literature, detailing how the regulation of a select class of lipid metabolic reactions are entwined with influences from the TME.

Just as diverse TME features regulate cancer cell lipid metabolism, lipid availability in the TME alters numerous metabolic pathways, beyond lipid synthesis and catabolism. Still, relatively little is known about how lipid availability impacts tumor metabolism, even though lipids are one of the most abundant and dynamic classes of metabolites in circulation (Dallmann et al., 2012; Quehenberger and Dennis, 2011; Quehenberger et al., 2010). As such, in the second half of this Review, we explore recent studies demonstrating that lipid availability affects disparate cellular metabolic processes in cancers. By discussing this literature, we aim to highlight how lipid availability has ‘ripple’ effects on the entire metabolic network due to the dense and interconnected nature of cell metabolism. Given the strongly entwined interactions between the TME and cellular metabolism, we also discuss experimental tools and approaches that are needed to integrate disparate areas of TME research and metabolic biology, to enable researchers to better understand how the TME influences cancer cell metabolic phenotypes and targets.

## Metabolites and stromal cells in the TME regulate lipid metabolism in cancer cells

**Fig. 1.**
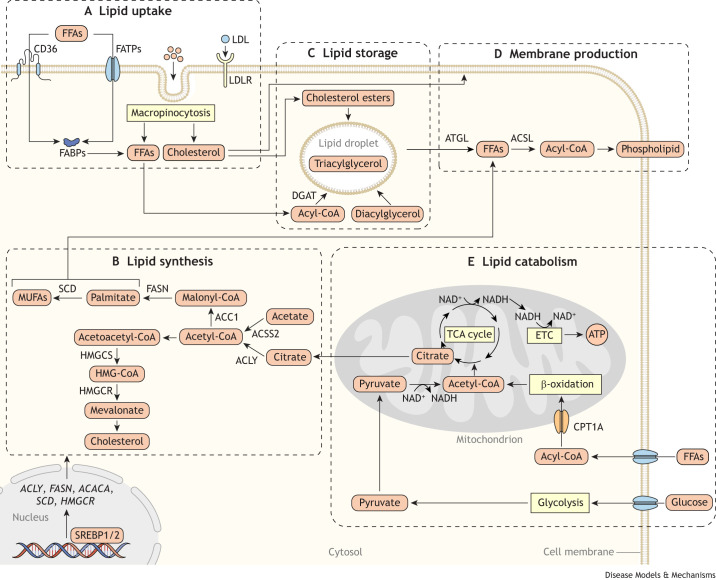
**Cancer cell lipid acquisition and utilization pathways.** (A) Cells take up lipids from the environment using several transport mechanisms. Cancer cells can use fatty acid transport proteins (FATPs), fatty acid-binding proteins (FABPs), cluster determinant 36 (CD36) and low-density lipoprotein (LDL) receptor (LDLR) to take up lipids from the environment. Additionally, cancer cells can take up bulk extracellular material via macropinocytosis, which provides a non-receptor-mediated pathway for cancer cells to acquire lipids. (B) Cells can also synthesize fatty acids and sterols from acetyl coenzyme A (CoA) using *de novo* synthesis metabolic pathways. The expression of enzymes involved in lipid *de novo* synthesis is transcriptionally regulated by the sterol regulatory element-binding protein (SREBP1 and SREBP2) transcription factors. Synthesizing lipids from acetyl-CoA consumes a considerable amount of cellular nicotinamide adenine dinucleotide phosphate (NADPH) pools, as fatty acid synthase (FASN), 3-hydroxy-3-methyl-glutaryl (HMG)-CoA reductase (HMGCR), stearoyl-CoA desaturase (SCD) and downstream sterol synthesis enzymes all use NADPH as a cofactor. Lipid synthesis also requires nicotinamide adenine dinucleotide (NAD^+^) to convert mitochondrial pyruvate into acetyl-CoA, which is processed to lipogenic citrate. Thus, via these shared co-factors, lipid synthesis pathways can interact with numerous other metabolic pathways in the cell. (C) Cancer cells can store both fatty acids and sterols as neutral lipids in lipid droplets for later mobilization and use. (D) Cancer cells need sterols and phospholipids, which are the building blocks for the production of cellular membranes. Fatty acids acquired through synthesis and uptake can be charged with CoA by acyl-CoA synthetase long-chain (ACSL) enzymes to allow for further synthesis of complex membrane lipids such as phospholipids. Sterols can also be synthesized *de novo* or acquired via uptake. Thus, cancer cells can produce membrane precursors by either *de novo* synthesis or by uptake from the environment. (E) Cancer cells can catabolize fatty acids to generate reducing equivalents and acetyl-CoA, both of which are used by the electron transport chain (ETC) to produce adenosine triphosphate (ATP). Abbreviations: ACC1, acetyl-CoA carboxylase 1, encoded by *ACACA*; *ACLY*, gene encoding ATP-citrate synthase; ACSS2, acyl-CoA synthetase short-chain family member 2; ATGL, adipose triglyceride lipase (also known as PNPLA2); CPT1A, carnitine palmitoyltransferase 1A; DGAT, diacylglycerol *O*-acyltransferase; FFA, free fatty acid; HMGCS, HMG-CoA synthase (encoded by *HMGCS1*); MUFA, monounsaturated fatty acid; NADH, reduced form of NAD^+^; TCA, tricarboxylic acid.

Lipid metabolism is the set of cellular metabolic processes that facilitate the acquisition and utilization of sterols (see Glossary, Box 1), phospholipids (Box 1) – which are important in membrane synthesis, and neutral lipids (Box 1) – which are critical for storage (Chandel, 2021; Harayama and Riezman, 2018; Yoon et al., 2021). These lipid species are essential for many processes that maintain cellular homeostasis. Cells use fatty acids (Box 1), sterols and complex lipids (Box 1) as building blocks for cell membranes during cell proliferation (Fig. 1D). Cells can also catabolize fatty acids from the circulation or phospholipids to produce energy in the form of adenosine triphosphate (ATP) via mitochondrial β-oxidation (Fig. 1E). Finally, nearly all healthy and malignant cells store sterols and fatty acids in specialized organelles called lipid droplets that act as reservoirs for lipids (Olzmann and Carvalho, 2019; Tirinato et al., 2017; Walther and Farese, 2012; Zadoorian et al., 2023) (Fig. 1C). Although there are no quantitative studies on the partition of lipids in cancer cells into these different pathways, there is increasing evidence that how cancer cells acquire and utilize lipids is influenced by the TME. The metabolism of lipid species such as membrane phospholipids and sphingolipids (Box 1) is often altered in cancers (Cheng et al., 2016; Ogretmen, 2018). However, TME regulation of the metabolism of these lipids is largely unexplored. In this section, we discuss how the TME regulates the choice between different lipid acquisition and utilization pathways in cancer. We focus specifically on the metabolic pathways related to fatty acid and cholesterol species that are required for tumors to meet their metabolic requirements of growth, and for which TME regulation has been the most extensively studied (Fig. 1).

**Box 1. Glossary**

**Acylcarnitines:** molecules formed by the conjugation of acyl-coenzyme A species with carnitine by the enzyme carnitine palmitoyltransferase 1 (CPT1). Fatty acid translocation into the mitochondria for β-oxidation requires CPT1-mediated transfer of the fatty acid to form the acylcarnitine. Thus, formation of acylcarnitines is critical for catabolism of lipid species.

**Adipokine:** a cytokine secreted by adipose tissue. These signaling molecules regulate various systemic functions including appetite, fat storage and glucose metabolism.

**Complex lipids:** lipids with multiple fatty acyl groups attached to a glycerol or sphingoid backbone. These lipids can also contain a headgroup, a non-fatty acyl substituent on the glycerol or sphingoid backbone. Examples of complex lipids include phospholipids and sphingolipids that are major constituents of cellular membranes, as well as neutral lipids such as triglycerides.

**Desmoplasia:** the growth of fibrous tissue around a site of an insult to a tissue – in the case of this Review, around a tumor.

**Fatty acids:** carboxylic acids with an aliphatic hydrocarbon chain. The hydrocarbon chain can be either saturated or unsaturated. Fatty acids are use to produce the fatty acyl side chains in complex lipids. Fatty acids can also be catabolized to generate energy.

**Ferroptosis:** a mechanism of cell death driven by lipid peroxidation in cellular membranes. Polyunsaturated fatty acids are particularly prone to oxidation via Fenton chemistry, which requires iron. Lipid hydroperoxides can be reduced by a variety of mechanisms in the cell, including glutathione-dependent peroxidase (GPX4). Thus, ferroptotic sensitivity in cells is regulated by the levels of polyunsaturated fatty acids, iron and glutathione in cells.

**Lysolipid:** a lipid with a glycerol or sphingoid backbone bound to one fatty acyl tail and a head group containing a phosphate. These lipids serve important signaling functions by binding G protein-coupled receptors. The most commonly studied lysolipids in tumors include sphingosine-1-phosphate and lysophosphatidic acid.

**Macropinocytosis:** a type of endocytosis in mammalian cells that is actin-driven and leads to the cellular ingestion of extracellular cargo. Macropinocytic vesicles are absorbed by the lysosome, leading to the breakdown of macromolecules to component precursors for use by the cell in biosynthetic processes.

**Neutral lipid:** lipids without any charge that are highly hydrophobic. The most common neutral lipids are triglycerides, which are composed of three fatty acyl tails bound to a glycerol backbone. Neutral lipids are primarily stored in lipid droplets that serve as important stores of fatty acids and sterols.

**Omentum:** a fold in the peritoneum that supports the stomach and liver, as well as other abdominal structures. Importantly for the studies referenced in this Review, the omentum is rich in adipocytes.

**One-carbon metabolism:** interlinked metabolic reactions in the serine/glycine, folate and methionine metabolic pathways that generate methyl groups (one-carbon units) used for the synthesis of methylated nucleotides, amino acids and phospholipids.

**Phospholipid:** a lipid with a glycerol backbone bound to one or two fatty acyl tails and a head group containing a phosphate. These lipids are the primary component of cellular plasma and organellar membranes.

**Sphingolipid:** a lipid with a sphingoid backbone with a fatty acyl tail. Sphingolipids may also contain various headgroups. They are an essential component of the plasma membrane.

**Pancreatic stellate cells:** a subclass of fibroblasts found in the pancreas that play a critical role in pancreatic fibrosis by synthesizing and depositing extracellular matrix.

**Sterol:** a lipid alcohol with a backbone of four carbon rings that can be modified to various species including cholesterol, the most abundant sterol in mammalian cells. Sterols play important roles in forming cellular membranes, and certain sterols (e.g. hormones and bile acids) have important signaling functions.

We want to highlight two key features of lipid metabolism. First, tumors broadly have two lipid acquisition pathways. Tumors can acquire the sterols, fatty acids and complex lipids needed for growth via *de novo* synthesis (Fig. 1B) from the precursor acetyl coenzyme A (CoA) or via uptake from the environment through fatty acid and sterol uptake systems (Fig. 1A). Although *de novo* lipid synthesis is restricted to select healthy tissues (Hollands and Cawthorne, 1981), many tumor cells express lipid synthesis enzymes and produce lipids *de novo*, at least in cell culture models (Menendez and Lupu, 2007; Röhrig and Schulze, 2016). How cells acquire lipids, whether by synthesis or uptake, is tightly regulated and linked to the interactions between the cell and its environment. Thus, the mode of lipid acquisition in cancer cells is tightly coupled to the TME. Second, lipid homeostasis requires lipid classes to be balanced in the cell. For example, having a balance of saturated and unsaturated fatty acids is essential for processes that involve membrane function, such as cellular signaling. A failure to maintain a healthy ratio of saturated and unsaturated fatty acids results in cell death (Liang et al., 2022; Sunshine and Iruela-Arispe, 2017; Volmer et al., 2013). Thus, cancers must tightly control and maintain the balance of lipid stoichiometry, despite challenges from TME stressors. Here, we highlight how lipid acquisition and utilization changes in cancer cells in response to TME to enable tumors to maintain lipid homeostasis despite the metabolic challenges of the TME.

### Hypoxia constrains tumor lipid metabolism

**Fig. 2.**
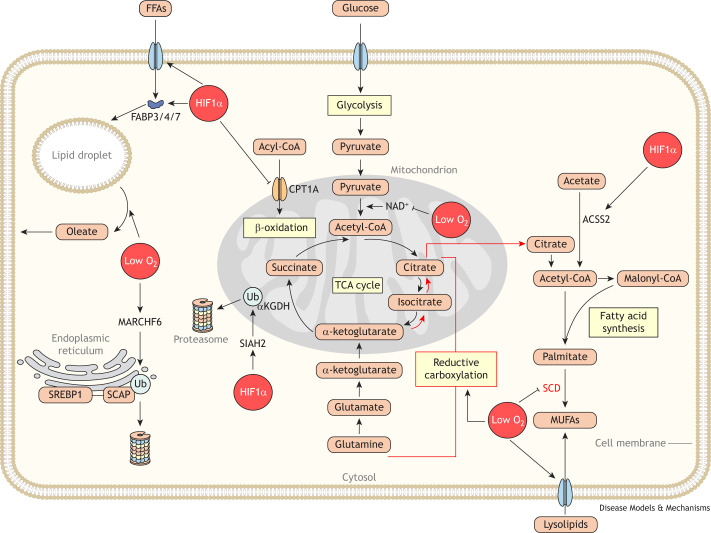
**Hypoxia changes lipid metabolism in cancer cells.** Hypoxia alters lipid metabolism in cancer cells both via substrate limitation, as oxygen (O_2_) is required in several lipid metabolic reactions, and via oxygen sensing signaling pathways such as hypoxia-inducible factor 1-α (HIF1α). At the substrate level, limited oxygen directly affects the activity of desaturase enzymes, such as stearoyl coenzyme A (CoA) desaturase (SCD). Thus, hypoxia limits the *de novo* synthesis of monounsaturated fatty acids (MUFAs). In response, cancer cells uptake lysolipids and release MUFAs from lipid droplets to provide oleate to cell membranes. Low oxygen tension also limits glucose-derived fatty acid synthesis by preventing the regeneration of nicotinamide adenine dinucleotide (NAD^+^). Hypoxia-driven limitation of NAD^+^ triggers the reductive use of glutamine as a carbon source for fatty acid synthesis in cancer cells. The red bracket indicates the pathway for reductive carboxylation of glutamine to citrate for *de novo* fatty acid synthesis. Several oxygen-sensitive signaling pathways also regulate cancer cell lipid metabolism under hypoxic conditions. HIF1α stabilization under hypoxia promotes lipid storage by enhancing lipid uptake through the fatty acid-binding proteins 3, 4 and 7 (FABP3/4/7) and decreasing β-oxidation of stored lipids by inhibiting carnitine palmitoyltransferase 1A (CPT1A). HIF1α facilitates acetate-derived lipid synthesis by promoting the expression of acyl-CoA synthetase short-chain family member 2 (ACSS2), which converts acetate into acetyl-CoA for cytosolic lipid synthesis. HIF1α also contributes to reductive carboxylation of glutamine by enhancing expression of the E3 ubiquitin ligase SIAH2, which ubiquitinates and ultimately degrades α-ketoglutarate dehydrogenase (αKGDH), ensuring that α-ketoglutarate is processed through the reductive side of the tricarboxylic acid (TCA) cycle. Lipid synthesis is also limited under oxygen-limited conditions by the membrane-associated ring-CH-type finger 6 (MARCHF6) oxygen-sensing ubiquitin ligase. MARCHF6 is active under hypoxia and triggers the degradation of SCAP–SREBP complex. Abbreviations: FFA, free fatty acid; SCAP, SREBP cleavage-activating protein; SREBP1, sterol regulatory element-binding protein 1; TCA, tricarboxylic acid; Ub, ubiquitin.

Many tumors have limited perfusion due to pathological angiogenesis and structural stresses on blood vessels, driven by uncontrolled growth and desmoplasia (Box 1) (DuFort et al., 2016; Fukumura et al., 2010; Jain, 2013; Jain et al., 2014; Olive et al., 2009). Limited perfusion results in reduced oxygen delivery, making hypoxia a key feature of many tumors (Challapalli et al., 2017; Wilson and Hay, 2011). Below, we discuss studies that demonstrate how hypoxia in the TME influences cellular lipid metabolism (Fig. 2).

Several enzymes that metabolize lipids require oxygen as a cofactor. For example, fatty acid desaturases, such as the enzyme stearoyl-CoA desaturase (SCD), utilize oxygen to catalyze the conversion of saturated fatty acids to unsaturated fatty acids and maintain a homeostatic balance between these fatty acids (Flowers and Ntambi, 2008; Paton and Ntambi, 2009; Sen et al., 2023). Thus, TME hypoxia reduces SCD activity (Agarwala et al., 2023; Kamphorst et al., 2013; Paton and Ntambi, 2009; Young et al., 2013) and constrains the ability of cancer cells to maintain a balance between unsaturated and saturated fatty acids.

Hypoxic cancer cells use two lipid metabolic adaptations to cope with limited desaturase activity. First, hypoxia enhances the uptake of exogenous unsaturated fatty acids and lysolipids (Box 1) in both normal and cancer cells (Bensaad et al., 2014; Kamphorst et al., 2013; Krishnan et al., 2009). This enables hypoxic cancer cells to acquire the unsaturated fatty acids that are needed to maintain a balance between saturated and unsaturated fatty acid levels, thus maintaining lipid homeostasis. Additionally, hypoxia triggers the release of monounsaturated fatty acids (MUFAs) from lipid droplets to compensate for loss of SCD activity in cancer cells. The catabolism of lipid droplets allows hypoxic cancer cells to maintain balanced fatty acid saturation when SCD activity is limited, facilitating cancer cell growth in the TME (Ackerman et al., 2018). Thus, hypoxia, by limiting a single cofactor required for lipid metabolic enzymes, can substantially alter how cancers acquire the unsaturated fatty acids that are required for lipid homeostasis.

In addition to directly regulating metabolic enzyme activity, hypoxia also indirectly impacts lipid metabolism in tumors. Oxygen serves as the terminal electron acceptor in the electron transport chain (ETC). In hypoxic conditions, the regeneration of nicotinamide adenine dinucleotide (NAD^+^) is limited due to reduced electron transport and concomitant loss of NADH oxidation (Birsoy et al., 2015; Sullivan et al., 2015). The reduction in NAD^+^ availability perturbs lipid metabolic reactions that require NAD^+^ as a cofactor. One such reaction is the oxidative production of citrate, a critical precursor of acetyl-CoA needed for fatty acid synthesis. Thus, citrate production and lipid synthesis are limited in hypoxic cells (Fan et al., 2013; Li et al., 2022; Wise et al., 2011).

Hypoxic cancer cells therefore require adaptations to overcome this restricted oxidative production of citrate. For example, they can produce citrate reductively by using glutamine as a carbon source (Fendt et al., 2013; Metallo et al., 2011; Mullen et al., 2011; Wise et al., 2011). However, reductive citrate production is unlikely to be sufficient to support *de novo* fatty acid synthesis (Li et al., 2022). Hypoxic cancer cells also adapt to reduced synthetic capacity by acquiring exogenous fatty acids (Li et al., 2022) or by synthesizing acetyl-CoA from other carbon sources taken up from outside of the cell. These sources include acetate (Gao et al., 2016), a metabolite that is increased in the TME of some tumors (Murthy et al., 2024), or citrate (Kumar et al., 2021). Thus, hypoxia indirectly constrains cancer cell fatty acid synthesis by limiting the ETC, which forces tumors to rely on alternative means of acquiring fatty acids.

In addition to regulating lipogenic substrate availability, hypoxia also regulates lipid metabolism by activating oxygen-sensing pathways, which regulate lipid metabolic reactions. For instance, hypoxia-sensing pathways regulate both fatty acid and sterol synthesis. Recent work has shown that sterol regulatory element-binding protein 2 (SREBP2, encoded by *SREBF2*), a key transcriptional regulator of sterol synthesis enzymes (Brown and Goldstein, 1997), is ubiquitinated and degraded by the hypoxia-responsive, endoplasmic reticulum-resident ubiquitin ligase named membrane-associated ring-CH-type finger 6 (MARCHF6) (Dickson et al., 2023) (Fig. 2). Interestingly, SREBP1 (encoded by *SREBF1*), a closely related protein that regulates fatty acid metabolism (Shimano and Sato, 2017), is not regulated by MARCHF6, suggesting that sterol and fatty acid metabolism are differentially regulated by hypoxia. Notably, the MARCHF6-mediated degradation of SREBP2 does not occur in tumor-infiltrating myeloid cells, in which hypoxia instead promotes SREBP2 processing by promoting Golgi–endoplasmic reticulum fusion (Nakahara et al., 2023). This suggests that different cell types experiencing hypoxia have different lipid metabolic responses.

The same hypoxia-responsive signaling pathways that constrain lipid synthesis also facilitate metabolic adaptations to decreased lipid synthesis. As discussed above, hypoxic cancer cells adapt to inhibited lipid synthesis in three main ways: (1) through the reductive generation of citrate for lipid synthesis; (2) through the uptake of exogenous acetate or citrate for lipid synthesis; and (3) through the uptake of exogenous lipids, circumventing lipid synthesis entirely. Hypoxia-sensing pathways contribute to all three of these adaptive mechanisms. For example, the hypoxia-stabilized transcription factor hypoxia-inducible factor 1-α (HIF1α, encoded by *HIF1A*) promotes the ubiquitination and degradation of the α-ketoglutarate dehydrogenase (αKGDH) complex by the E3 ubiquitin ligase SIAH2 (Sun and Denko, 2014) (Fig. 2). This degradation inhibits glutamine oxidation and promotes the reductive generation of citrate and lipid synthesis under hypoxic conditions (Sun and Denko, 2014). Additionally, HIF1α promotes the expression of acyl-CoA synthetase short-chain family member 2 (ACSS2), which converts acetate to cytosolic acetyl-CoA. Thus, HIF1α activation of ACSS2 allows cancer cells to use acetate lipid synthesis in hypoxic conditions when oxidative production of acetyl-CoA may be limited (Comerford et al., 2014; Schug et al., 2015) (Fig. 2). Hypoxia-responsive signaling also regulates lipid uptake in cancer cells. HIF1α increases cellular lipid uptake by inducing the expression of fatty acid-binding proteins (FABPs) 3 and 7 (FABP3 and FABP7) in glioblastoma, which are both required for cancer cell survival in hypoxic conditions (Bensaad et al., 2014). HIF1α signaling also promotes lipid uptake by increasing the expression of lipoprotein receptors in cancer cells (Shen et al., 2012). In addition to HIF1α, hypoxia activates other pathways that promote lipid accumulation. Hypoxia increases lipid uptake and FABP4 levels in ovarian cancer cells by downregulating the FABP4 inhibitory microRNA miR-409-3p (Gharpure et al., 2018). Thus, hypoxia-sensing pathways enable cells to switch from oxidative *de novo* lipid synthesis to alternative pathways of lipid acquisition that are not limited by oxygen availability.

Lastly, in addition to regulating lipid synthesis and uptake in cancer cells, hypoxia also regulates the metabolic utilization of lipids in cancer cells. HIF1α activity downregulates the expression of carnitine palmitoyltransferase 1A (CPT1A) and medium- and long-chain acyl-CoA dehydrogenases, key enzymes for the import of mitochondrial fatty acids and for the β-oxidation of lipids, thus limiting fatty acid catabolism in cancer cells (Du et al., 2017; Huang et al., 2014; Miess et al., 2018). HIF signaling instead promotes increased storage of fatty acids in lipid droplets, both by increasing the transcription of lipid droplet proteins (Qiu et al., 2015) and by inhibiting enzymes that hydrolyze lipids from droplets (Zhang et al., 2017b). Importantly, redirecting fatty acids from catabolism towards storage in lipid droplets is critical for tumor growth (Du et al., 2017; Qiu et al., 2015; Zhang et al., 2017b). Therefore, oxygen-sensing pathways allow tumors to adapt to hypoxia by regulating the metabolic fates of lipids in cancer cells.

When considered together, a key theme emerges from these studies: TME hypoxia constrains *de novo* lipogenesis in cancers. In response, hypoxia-sensing pathways enable tumors to cope with this constraint by activating alternative pathways, such as lipid uptake, to allow tumors to acquire the lipids they need for their homeostasis and growth.

### Amino acid availability in the TME affects cancer lipid metabolism

**Fig. 3.**
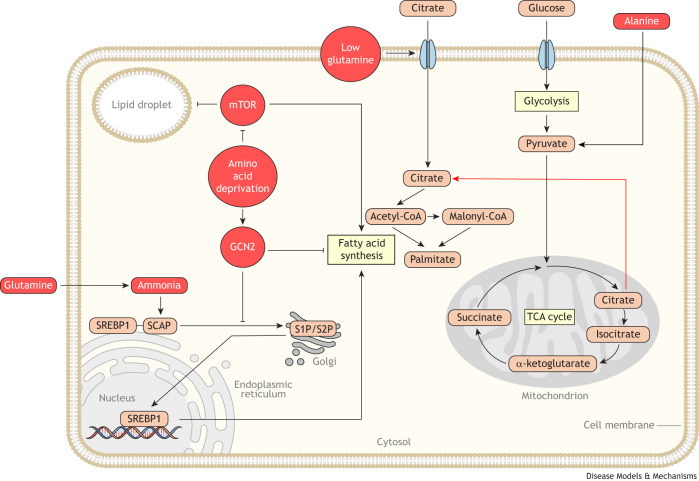
**Amino acid availability constrains lipid metabolism in cancer cells.** Amino acid availability alters the metabolic routes that cancer cells use to acquire lipids. Amino acid-replete conditions promote *de novo* lipid synthesis. For example, glutamine-replete conditions enhance lipid synthesis by stimulating SCAP–SREBP release from the endoplasmic reticulum through ammonia post-translational modifications from glutamine. Similarly, alanine availability promotes lipid synthesis by serving as a source of acetyl coenzyme A (CoA). Starvation of amino acids downregulates lipid synthesis through the general control nonderepressible protein 2 (GCN2) and mammalian target of rapamycin (mTOR) amino acid-sensing pathways. GCN2 activation under amino acid deprivation inactivates SREBP transcription factors, leading to decreased fatty acid synthesis. When mTOR is inactivated under amino acid starvation, fatty acid synthesis decreases and fatty acid storage in lipid droplets increases. Amino acid starvation can also alter how cells acquire biomass for lipid synthesis. For example, under low glutamine conditions, cells increase uptake of citrate for use in lipid synthesis. Abbreviations: S1P/S2P, site-1 protease (encoded by *MBTPS1*) and site-2 protease (encoded by *MBTPS2*); SCAP, SREBP cleavage-activating protein; SREBP1, sterol regulatory element-binding protein 1.

Limited vascular perfusion and altered metabolic activity of both malignant and stromal cells can lead to pathophysiological concentrations of amino acids in the TME (Apiz Saab et al., 2023; Fukumura et al., 2010; Menjivar et al., 2023). Variability in amino acid abundance influences lipid metabolism in two ways: directly, as amino acids can be substrates for lipid metabolic reactions, and indirectly, through cellular programs that alter cell signaling in response to amino acid abundance. Below (and in Fig. 3), we discuss how amino acid availability in the TME regulates lipid metabolism in tumors.

#### Amino acids as a carbon source for lipogenesis

Glutamine (Fendt et al., 2013; Metallo et al., 2011; Mullen et al., 2011; Wise et al., 2011), as discussed previously, can be used as a carbon source to produce lipogenic acetyl-CoA when glucose-derived citrate is depleted. In pancreatic cancer, alanine, which is abundant in the pancreatic TME due to its excretion by pancreatic stromal stellate cells (Box 1), can also be a source of acetyl-CoA for lipogenesis (Sousa et al., 2016). Pancreatic cancers can also catabolize branched-chain amino acids (Zhu et al., 2020) to support lipogenesis (Lee et al., 2019), a pathway similarly used by adipocytes to fuel lipogenesis (Green et al., 2016). Beyond fatty acid synthesis, cancer cells can also use amino acids for complex lipid synthesis. The amino acid serine, for example, is necessary for sphingolipid synthesis. In colorectal cancer, a reduction of serine in the TME leads to the decreased production of canonical sphingolipids (Gao et al., 2018; Muthusamy et al., 2020). Importantly, the enzyme that uses serine as a substrate, serine palmitoyltransferase (SPT), is promiscuous and can use alanine as a substrate to produce 1-deoxysphingolipid when serine levels are low (Lone et al., 2019). Under conditions of serine deprivation, colorectal tumors produce toxic levels of 1-deoxysphingolipid that slow tumor growth (Muthusamy et al., 2020). Thus, by virtue of serving as substrates in multiple lipid metabolic pathways, amino acids in the TME directly impact tumor lipid metabolism.

Amino acid abundance also indirectly affects cellular lipid metabolism through amino acid-sensing signaling pathways that regulate lipid metabolism (Efeyan et al., 2015; Palm and Thompson, 2017; Torrence and Manning, 2018). In particular, mammalian target of rapamycin (mTOR), general control nonderepressible protein 2 (GCN2, encoded by *EIF2AK4*) and SREBP can all sense cellular amino acid abundance and alter lipid metabolism.

#### mTOR

mTOR is an amino acid-sensing protein complex that regulates lipid metabolism (Caron et al., 2015; Lamming and Sabatini, 2013; Ricoult and Manning, 2013). In amino acid-replete conditions, mTOR complex 1 (mTORC1) is active and drives lipid biogenesis by activating SREBP transcription, processing and trafficking (Caron et al., 2015; Lamming and Sabatini, 2013; Ricoult and Manning, 2013). However, in solid tumors that have decreased amino acid availability, mTORC1 can be inactivated (Bielska et al., 2022; Palm et al., 2015). Thus, amino acid stress in the TME likely dampens lipogenesis in tumors by suppressing mTOR activity, although this remains to be assessed. Interestingly, mTOR inactivation appears to be adaptive for tumors and is required for cell survival under nutrient stress (Bielska et al., 2022; Palm et al., 2015).

Decreased mTOR activity also aids cells that are coping with nutrient stress by changing the way cells utilize lipids. For example, amino acid starvation leads to increased fat storage in lipid droplets in an mTOR-dependent manner (Nguyen et al., 2017). Increased fat storage is contingent on the lipid droplet regulators diacylglycerol *O*-acyltransferase 1 (DGAT1) (Nguyen et al., 2017) and hypoxia-inducible lipid droplet-associated protein (HILPDA) (VandeKopple et al., 2019) and prevents excessive fatty acid β-oxidation. When cells are unable to store lipids in droplets under nutrient starvation, acylcarnitines (Box 1) accumulate, triggering mitochondrial dysfunction and cell death (Nguyen et al., 2017). This is reflected in xenograft tumors of human colorectal cancer cells in mice, in which the ablation of HILPDA decreased tumor growth and lipid droplet accumulation (VandeKopple et al., 2019). Thus, colorectal tumors must store lipids in lipid droplets rather than oxidize them to survive amino acid deprivation in the TME (VandeKopple et al., 2019). This implies that mTOR suppression enables tumors to survive in the amino acid-deprived TME by altering lipid utilization.

#### GCN2

GCN2 is an integrated stress response kinase that senses amino acid deprivation via uncharged tRNAs and stalled ribosomes. During amino acid starvation in tissues, GCN2 suppresses lipid synthesis by inhibiting the expression of SREBP and of SREBP target genes (Dudek and Semenkovich, 1995; Guo and Cavener, 2007). Interventions that lower TME amino acid availability suppress SREBP activity and lipid synthesis in tumors in a GCN2-dependent manner (Xiao et al., 2016). Thus, GCN2 signaling might be a critical pathway by which amino acid abundance in the TME regulates tumor lipid synthesis.

#### SREBP

Lastly, SREBP transcription factors themselves sense the availability of specific amino acids. For example, glutamine activates lipogenesis in tumors by activating SREBP cleavage-activating protein (SCAP) (Cheng et al., 2022). Interestingly, the catabolism of glutamine to ammonia is required for SREBP activation, indicating that SREBP responds to ammonia availability as well, which is elevated in the TME of solid tumors (Spinelli et al., 2017).

Altogether, these studies demonstrate that amino acid deprivation perturbs lipogenesis in cancer cells. Amino acid-sensing programs also change the way cancer cells utilize lipids to continue meeting the metabolic needs of tumors, facilitating continued growth.

### Glucose availability in the TME affects cancer lipid metabolism

**Fig. 4.**
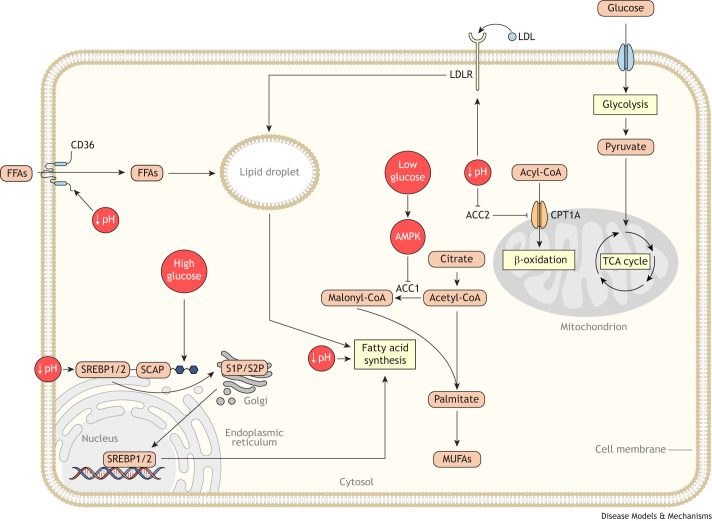
**Glucose availability and acidity alter lipid metabolism in cancer cells.** Both glucose abundance and acidity alter lipid metabolism in cancer cells. Glucose abundance promotes lipid accumulation by enhancing SREBP-mediated lipid synthesis, which occurs through glycosylation and activation of SCAP, which promotes SREBP maturation. In contrast, glucose depletion decreases lipid synthesis. Glucose starvation leads to AMP-activated protein kinase (AMPK) activation, which inhibits acetyl-CoA carboxylase 1 (ACC1) and *de novo* lipid synthesis. Low pH also alters lipid metabolism in cancer cells. Acidity increases cluster determinant 36 (CD36)- and low-density lipoprotein (LDL) receptor (LDLR)-mediated lipid uptake and increases fatty acid synthesis by promoting SREBP processing. Thus, acidity promotes lipid acquisition in cancer cells. Acidity also regulates the utilization of lipids. Low pH environments increase β-oxidation of lipids by inhibiting acetyl-CoA carboxylase 2 (ACC2), which restrains β-oxidation. Abbreviations: CoA, coenzyme A; CPT1A, carnitine palmitoyltransferase 1A; FFA, free fatty acid; MUFAs, monounsaturated fatty acids; S1P/S2P, site-1 protease and site-2 protease; SCAP, SREBP cleavage-activating protein; SREBP1/2, sterol regulatory element-binding proteins 1 and 2; TCA, tricarboxylic acid.

Some cancers have limited glucose in their TME (Gullino et al., 1965; Helmlinger et al., 2002; Ho et al., 2015; Nwosu et al., 2023). Below (and in Fig. 4), we discuss how limited glucose in the TME impacts tumor lipid metabolism.

In cancer cells, glucose limitation triggers many nutrient-sensing pathways that interface with lipid metabolism. One such pathway is SREBP transcriptional activity. Glucose mediates SCAP glycosylation and activation, facilitating the activation of SREBP and downstream signaling (Cheng et al., 2015). Thus, glucose availability dictates the transcription of lipogenic genes through the SREBP–SCAP axis. Glucose deprivation also influences lipid metabolism by lowering cellular ATP levels and activating AMP-activated protein kinase (AMPK) (Hardie et al., 2016; Herzig and Shaw, 2018; Lin and Hardie, 2018). AMPK inhibits cellular fatty acid synthesis by phosphorylating and inhibiting acetyl-CoA carboxylase 1 (ACC1, encoded by *ACACA*), limiting the conversion of the lipogenic substrate acetyl-CoA to malonyl-CoA (Hardie et al., 2016; Herzig and Shaw, 2018; Lin and Hardie, 2018). Notably, xenograft tumors of both human lung and breast cancer cells in mice rely on AMPK signaling and downregulated lipid synthesis to survive glucose deprivation (Jeon et al., 2012). If AMPK is perturbed, continued fatty acid synthesis exhausts cellular NADPH pools, resulting in impaired regeneration of reduced glutathione and making cells sensitive to redox stress. Thus, glucose deprivation in tumors, similar to amino acid deprivation and hypoxia, suppresses lipid synthesis in cancer cells. This suppression preserves cellular NADPH pools, which are essential for proliferating tumors.

### Tumor acidity impacts lipid metabolism

The TME is often acidic due to the high levels of metabolic activity in the tumor and limited perfusion, which is needed to remove acidic waste (Corbet and Feron, 2017; Swietach et al., 2023; Webb et al., 2011). In numerous studies, TME acidity has been shown to induce the accumulation of lipid droplets in cancer cells, driven by both increased lipid synthesis and increased lipid uptake, depending on the cell type (Corbet et al., 2020; Dierge et al., 2021; Ding et al., 2022; Menard et al., 2016; Nardi et al., 2019). In human colorectal and squamous cell carcinoma cells (Corbet et al., 2020; Dierge et al., 2021), lipid uptake via cluster determinant 36 (CD36) is required for acid-induced lipid droplet formation (Corbet et al., 2020; Dierge et al., 2021). In human glioblastoma cell lines (Menard et al., 2016), lipid droplet fatty acids come from lipoprotein uptake (Menard et al., 2016). In contrast, in liver cancer cells, acid-induced lipid droplets form via increased fatty acid synthesis (Ding et al., 2022) and, in pancreatic cancer, acidity increases SREBP activity and sterol synthesis (Kondo et al., 2017).

Acid-driven lipid accumulation is functionally important to tumors. Acidity increases fatty acid β-oxidation by downregulating acetyl-CoA carboxylase 2 (ACC2), a negative regulator of β-oxidation (Corbet et al., 2016). The enhanced β-oxidation of lipids stored in lipid droplets is an important source of ATP, which is necessary for metastatic progression (Corbet et al., 2020). Inhibiting β-oxidation reverses the enhanced metastatic potential of cancer cells (Corbet et al., 2020). Thus, acidity in the TME regulates both lipid abundance and utilization in cancer cells (Fig. 4).

Notably, cancer cells in different contexts require lipid droplets for distinct purposes. As discussed previously, nutrient-deprived tumors require lipid droplets to sequester a toxic buildup of acylcarnitines (Nguyen et al., 2017; VandeKopple et al., 2019), whereas lipid droplet hydrolysis is required in cancer cells in acidic environments to produce energy via β-oxidation (Corbet et al., 2020). How lipid metabolism in cancer cells responds to environments that are both hypoxic and acidic remains to be determined.

### Stromal cells in the TME and tumor lipid metabolism

**Fig. 5.**
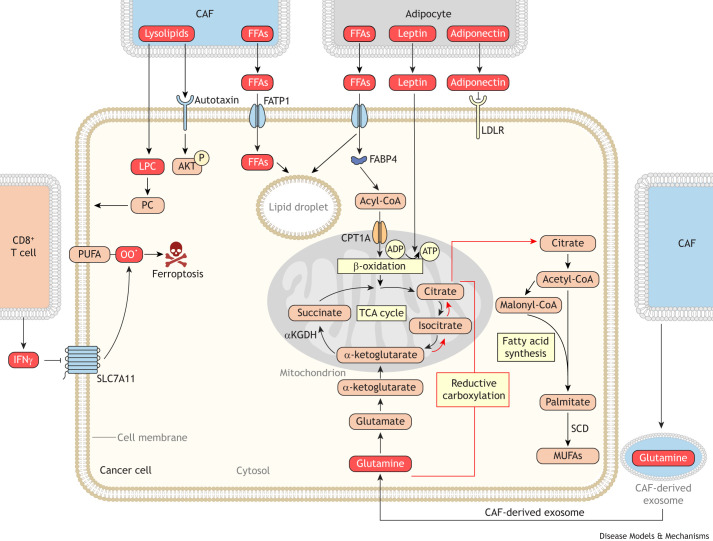
**Stromal cells in the tumor microenvironment alter lipid metabolism in cancer cells.** Stromal cells alter cancer cell lipid metabolism by providing lipids directly to cancer cells. Cancer-associated fibroblasts (CAFs) and adipocytes can provide fatty acids, sterols and lysophospholipids to cancer cells, which use these lipids for membrane production and for energy production through β-oxidation. Additionally, CAFs promote lipid synthesis by providing cancer cells with glutamine packaged in exosomes for use in reductive lipid synthesis. The red bracket indicates the pathway for reductive carboxylation of glutamine to citrate for *de novo* fatty acid synthesis. Stromal cells also alter lipid metabolism via cytokine and adipokine signaling. CD8^+^ T cells release interferon γ (IFNγ) in the tumor microenvironment, which triggers decreased SLC7A11 expression in cancer cells. Decreased SLC7A11 expression in cancer cells leads to increased lipid peroxidation (OO**^·^**) and ferroptotic cell death. Adiponectin release from adipocytes alters lipid metabolism in cancer cells by decreasing low-density lipoprotein receptor (LDLR)-mediated lipid uptake, whereas leptin signaling promotes β-oxidation in cancer cells. Abbreviations: αKGDH, α-ketoglutarate dehydrogenase; ADP, adenosine diphosphate; AKT, AKT serine/threonine kinase; ATP, adenosine triphosphate; CoA, coenzyme A; CPT1A, carnitine palmitoyltransferase 1A; FABP4, fatty acid-binding protein 4; FATP1, fatty acid transport protein 1; FFA, free fatty acid; LPC, lysophosphatidylcholine; MUFAs, monounsaturated fatty acids; PC, phosphatidylcholine; PUFA, polyunsaturated fatty acid; SCD, stearoyl-CoA desaturase; SLC7A11, solute carrier family 7 member 11; TCA, tricarboxylic acid.

Tumors can be thought of as complex ecosystems that contain both malignant and stromal cells that interact in intricate ways (de Visser and Joyce, 2023). In the following section, we discuss how stromal cells in the TME influence tumor lipid metabolism (Fig. 5).

#### Adipocytes

Adipocytes in the TME provide lipids to cancer cells. These lipids are consumed via β-oxidation in cancer cells in multiple cancer models, including mouse models of ovarian and breast tumors, and zebrafish models of melanoma (Mukherjee et al., 2020, 2023; Nieman et al., 2011; Wang et al., 2017; Zhang et al., 2018). In ovarian cancer, adipocytes produce fatty acids, which are taken up by FABP4-expressing cancer cells and oxidized into ATP (Mukherjee et al., 2020). Because ovarian tumor cells use fatty acids to produce ATP, ovarian cancer metastases preferentially home to adipocyte-rich sites such as the omentum (Box 1) (Mukherjee et al., 2020, 2023; Nieman et al., 2011). Interestingly, FABP4 expression also promotes carboplatin resistance in human ovarian cancer cells in culture and when xenografted in mice, indicating that adipocyte-induced FABP4 expression is critical to both growth and survival in the presence of chemotherapy in ovarian tumors (Mukherjee et al., 2020). Similarly, human breast cancer cells, both in culture and in murine xenograft tumors, take up adipocyte-derived free fatty acids, which can be stored in lipid droplets or oxidized for energy production (Wang et al., 2017). Disrupting this process by genetically perturbing the hydrolysis of fatty acids from lipid droplets or by pharmacologically inhibiting lipid oxidation inhibited breast cancer invasion in human cell lines and syngeneic mouse models of breast cancer (Wang et al., 2017). Lastly, melanoma invasion requires fatty acid transport protein (FATP)-mediated uptake of adipocyte-derived fatty acids in zebrafish models of breast cancer (Zhang et al., 2018). Altogether, these studies indicate that adipocyte-derived fatty acids are crucial for growth and metastatic progression in multiple models of diverse cancers.

Aside from providing fatty acids to tumor cells, adipocytes regulate cancer lipid metabolism by releasing adipokines (Box 1). A common adipokine to influence cancer growth is leptin, which is secreted by adipocytes and regulates systemic energy homeostasis. The leptin receptor (LEPR) is expressed in many tumors, making cancers primed to respond to adipocyte-derived leptin (de Candia et al., 2021). In both human breast cancer cell lines and genetically engineered mouse models of breast cancer, leptin exposure promotes fatty acid β-oxidation (Blanquer-Rosselló et al., 2016; Liu et al., 2019; Pham et al., 2021; Wang et al., 2018). Notably, human breast cancer cells exposed to leptin grew more aggressively than unexposed cells, indicating that adipocyte-derived leptin plays a role in driving tumor progression (Wang et al., 2018). Other adipokines, such as adiponectin, perturb breast tumor proliferation by inhibiting cholesterol uptake in genetically engineered mouse models of breast cancer (Liu et al., 2013). These findings show that the way in which adipokines influence tumor growth is complex. Future studies are therefore needed to determine how tumor and lipid metabolism respond to different adipocyte signals with diverging outcomes.

#### Cancer-associated fibroblasts

In multiple tumor types, stromal fibroblasts influence lipid metabolism by providing lipids and lipogenic precursors to cancer cells. In human melanoma and breast cancer cell lines, exposure to cancer-associated fibroblasts (CAFs) increases the lipid content of cancer cells. This requires both the secretion of lipids by fibroblasts and the transport of these lipids by FATPs in the cancer cells (Alicea et al., 2020; Lopes-Coelho et al., 2018). Interestingly, in both cancer types, CAF exposure increases FATP expression in the cancer cells, suggesting that CAFs not only provide lipids, but also secrete paracrine signaling factors to facilitate the uptake of these lipids (Alicea et al., 2020; Lopes-Coelho et al., 2018). Pharmacologically blocking FATP-mediated lipid uptake with FATP inhibitors in melanoma slows tumor progression and prevents resistance to targeted therapies (Alicea et al., 2020). This suggests that this metabolic interaction between CAFs and melanoma cells is crucial for disease progression. In co-culture with human pancreatic cancer cell lines, CAFs alter cancer lipid metabolism by secreting lysophosphatidylcholine (LPC) (Auciello et al., 2019). Pancreatic cancer cells use CAF-derived LPC to synthesize phosphatidylcholine (Auciello et al., 2019). CAF-secreted LPC also alters cellular signaling when hydrolyzed to lysophosphatidic acid in pancreatic tumors, which promotes oncogenic signaling and tumor progression through AKT serine/threonine kinase (AKT) activation (Auciello et al., 2019).

In addition to providing lipids directly to cancer cells, CAFs regulate tumor lipid metabolism by altering substrate availability for lipid synthesis in tumors. In prostate and pancreatic cancer cells, CAF-derived exosomes supply tumors with glutamine, glucose and other metabolites that can be used as carbon sources for lipid biosynthesis. In particular, cultured prostate and pancreatic cancer cells use glutamine from CAF-derived exosomes to synthesize lipids via reductive carboxylation (Zhao et al., 2016). Additionally, as discussed above, pancreatic tumor stellate cells provide cancer cells with alanine that can similarly be used for lipogenic reactions through its conversion to acetyl-CoA (Sousa et al., 2016).

#### Immune cells

There is increasing evidence that immune cells in the TME also regulate lipid metabolism in cancer cells. For example, immune signaling in the TME has recently been shown to constrain tumor progression by regulating lipid metabolism. In mouse models of ovarian cancer and melanoma, CD8^+^ T cells alter lipid metabolism in tumors by releasing interferon γ (IFNγ, encoded by *IFNG*) (Wang et al., 2019). In response to IFNγ, cancer cells downregulate SLC7A11 and SLC3A2 expression, which together mediate cysteine import in mammalian cells (Lewerenz et al., 2013). With cysteine import limited, cancer cells are unable to synthesize sufficient glutathione to reduce lipid peroxides (Wang et al., 2019). These lipid peroxides accumulate in IFNγ-exposed cancer cells and trigger ferroptosis (Box 1), a mode of cell death due to accumulation of oxidized lipids (Dixon and Olzmann, 2024; Jiang et al., 2021). Thus, immune signaling in the TME can potently affect cancer cell lipid metabolism. Further studies are necessary to fully understand how the complex cytokine signaling networks present in the TME affect tumor lipid metabolism.

In addition to regulating tumor lipid metabolism via cytokine signaling, it is possible that immune cells dictate lipid metabolism in cancer cells by directly altering lipid availability in the TME. Many TME-infiltrating immune cells rapidly import extracellular lipids (Herber et al., 2010; Ma et al., 2021; Masetti et al., 2022; Miska et al., 2022; Su et al., 2020; Wang et al., 2020; Xu et al., 2021). Furthermore, some immune cells release lipids in the TME. For example, tumor-infiltrating macrophages were found to release cholesterol in mouse models of ovarian and prostate cancer (El-Kenawi et al., 2021; Kaymak et al., 2022). Thus, immune cells may restrict TME levels of certain lipids, while increasing the availability of others. Further studies are necessary to understand how immune cells affect TME lipid availability and how such regulation impacts cancer cell lipid metabolism.

The studies discussed above investigate how lipid metabolism in cancer cells responds to isolated features of the TME. It is clear in these studies that different elements of the TME divergently regulate lipid metabolism in cancer cells. As cancers face multiple TME stresses simultaneously, future work is needed to examine how tumor lipid metabolism changes in response to multiple TME features. Nevertheless, a key finding that emerges from these studies is that most TME features suppress *de novo* lipid synthesis in cancer. Tumors instead utilize alternative lipid acquisition strategies, such as increasing lipid uptake from the environment or stromal cells. Thus, lipid uptake may be an underappreciated metabolic requirement of cancers.

Lastly, although we have focused this Review on how the TME impacts lipid metabolism in cancer cells, the TME also affects lipid metabolism in other cell types present in the TME. Notably, the TME affects lipid metabolism in immune cells, which affects immune control of tumors (Broadfield et al., 2021; Lou et al., 2022; Marelli et al., 2022; Vitale et al., 2019; Zhang et al., 2024). We refer readers to the following references for comprehensive reviews on how the TME affects immune cell lipid metabolism and function: Broadfield et al. (2021); Lou et al. (2022); Marelli et al. (2022); Vitale et al. (2019); and Zhang et al. (2024).

## Lipid availability in the TME regulates tumor metabolism

The studies discussed above show that constraints in the TME promote the reliance of tumors on exogenous lipids. As such, to understand tumor metabolism, we need to better understand lipid availability in the TME. Although we know less about lipid availability relative to other features of the TME, a small but growing body of work suggests that TME lipids are important regulators of cancer metabolism. Lipids influence not only tumor lipid metabolism, but also how cancers metabolize other substrates such as glucose. In this section, we review studies that show that lipids are important microenvironmental drivers of cancer metabolism and biology.

### TME lipid availability affects tumor lipid metabolism

**Fig. 6.**
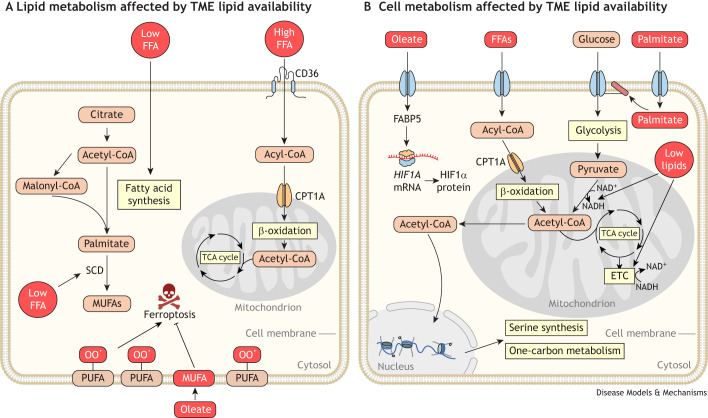
**Lipid availability alters cancer cell metabolism.** (A) Lipid availability in the tumor microenvironment (TME) regulates cancer lipid metabolism. High levels of lipids in the TME promote β-oxidation in cancer cells by providing fatty acids that are taken up by receptors such as cluster determinant 36 (CD36) for subsequent catabolism. Oleate-rich environments, such as the lymph to which metastasizing cancer cells are exposed, lead to increased incorporation of monounsaturated fatty acids (MUFAs) into the membranes of cancer cells. This renders cancer cells resistant to ferroptosis as MUFAs replace oxidation-prone polyunsaturated fatty acids (PUFAs) in cell membranes. In contrast, TMEs that are lipid deplete, such as those in the brain, trigger increased fatty acid synthesis in cancer cells. This change in the lipid acquisition mechanism allows cancer cells to continue to grow even in lipid-poor environments. (B) Lipid availability in the TME also affects metabolism of non-lipid species in cancer cells. Lipid availability serves as a cue that regulates cellular signaling pathways and the expression of metabolic genes to broadly affect cancer cell metabolism. For instance, exposure to oleate alters HIF1α translation in cancer cells by upregulating fatty acid-binding protein 5 (FABP5). Free fatty acids (FFAs) are also a source of acetyl coenzyme A (CoA) that alters histone acetylation, thus altering serine synthesis and one-carbon metabolism in cancer cells. Additionally, lipid availability affects metabolism by impacting nutrient uptake in cancer cells. Palmitate post-translationally modifies nutrient transporters, including glucose transporters. Thus, fatty acid availability increases glucose uptake and glycolysis in cancer cells. Finally, lipid availability impacts other metabolic pathways via the use of shared redox cofactors. For example, lipid deprivation increases lipid synthesis in cancer cells. Increased lipid synthesis consumes and lowers nicotinamide adenine dinucleotide (NAD^+^) levels in cancer cells. Cancer cells increase oxygen consumption to increase NAD^+^ regeneration through the electron transport chain (ETC) as a result. Abbreviations: CPT1A, carnitine palmitoyltransferase 1A; NADH, reduced form of NAD^+^; OO**^·^**, peroxidation; SCD, stearoyl-CoA desaturase; TCA, tricarboxylic acid.

Extracellular lipids are transported into the TME from the systemic circulation by carrier proteins, such as albumin and lipoproteins (Van der Horst et al., 2009). After entering the TME, cancer cells take up these lipids using lipid transporters (e.g. CD36 or FATPs) (Glatz et al., 2010) or lipoprotein receptors (Deng et al., 2022), or by macropinocytosis (Box 1) (Salloum et al., 2023) (Fig. 1A). Here, we discuss how lipid availability alters tumor lipid metabolism (Fig. 6A).

Lipid availability in the TME is dynamic. Several studies have found that modifying dietary fat intake changes lipid metabolism in tumors (Altea-Manzano et al., 2023; Goswami et al., 2023; Lien and Vander Heiden, 2019; Lien et al., 2021; Ringel et al., 2020). In mouse models of multiple cancers (Altea-Manzano et al., 2023; Mana et al., 2021; Pascual et al., 2017; Ringel et al., 2020), high-fat diets increase fatty acid concentrations in the TME (Altea-Manzano et al., 2023; Lien et al., 2021), thus enhancing fatty acid uptake and oxidation (Altea-Manzano et al., 2023; Mana et al., 2021; Pascual et al., 2017; Ringel et al., 2020). These diet-driven changes in lipid metabolism are required for tumor progression. Inhibiting lipid uptake and oxidation slows tumor progression in genetically engineered mouse models of breast and colorectal cancer, and metastasis in xenograft models of squamous cell carcinoma (Altea-Manzano et al., 2023; Mana et al., 2021; Pascual et al., 2017). Dietary interventions can also lower lipid availability in the TME. For example, caloric restriction lowers fatty acid levels in mouse models of pancreatic cancer. This forces pancreatic tumors to rely heavily on the *de novo* synthesis of fatty acids, particularly of MUFAs, for growth (Lien et al., 2021).

Lipid availability also changes as tumors metastasize. For example, in early metastatic spread, human melanoma cells injected into mice that invade the lymph are exposed to an environment enriched in MUFAs (Fig. 6A) (Ubellacker et al., 2020). MUFAs prevent lipid peroxidation and cell death by ferroptosis (Magtanong et al., 2019). As metastasizing melanoma cells incorporate these MUFAs into phospholipids in cellular membranes, they acquire resistance to ferroptosis, facilitating metastatic spread (Ubellacker et al., 2020). Lipid availability in metastatic sites can also influence disease progression. As discussed above, ovarian and melanoma mouse models of cancer that invade adipose-rich tissues adapt by taking up and using fatty acids from the lipid-rich metastatic TME (Mukherjee et al., 2020, 2023; Nieman et al., 2011; Wang et al., 2017; Zhang et al., 2018). Liver steatosis, in which lipids accumulate in hepatocytes, makes the liver more susceptible to metastatic colonization by breast cancer cells (Li et al., 2020).

Not every metastatic site is lipid rich. Notably, the brain microenvironment has very few exogenous lipids (Ferraro et al., 2021). Thus, brain metastases arising from murine xenograft breast tumor substantially upregulate *de novo* lipid synthesis (Ferraro et al., 2021; Jin et al., 2020). As a result, genetic perturbation of *de novo* lipid synthesis inhibits the growth of brain metastases in these mouse models of breast cancer, while the primary tumor remains unaffected.

These studies show that lipid availability in the TME is a key regulator of lipid metabolism in cancer cells. There is also emerging evidence that TME lipids are key regulators of lipid biology and function in immune cells. We refer readers interested in this topic to recent reviews on this topic (Corn et al., 2020; Goswami et al., 2023; Prendeville and Lynch, 2022). The following section highlights how lipid availability alters features of cancer cell metabolism beyond lipid metabolism, exhibiting metabolic ripple effects across the cancer cell metabolome.

### TME lipids alter non-lipid metabolism in cancer cells

As metabolic pathways are highly interconnected, changes in lipid availability have ripple effects that alter many metabolic pathways. As a result, lipid availability in cancer cells affects metabolic nodes beyond lipid metabolism, as we discuss below (Fig. 6B).

As mentioned above, oxidative metabolism in mitochondria is linked to lipid synthesis through the NAD^+^/NADH redox cofactor. Lipid synthesis, by consuming large amounts of acetyl-CoA, consumes a substantial portion of the cellular NAD^+^ pool (Li et al., 2022), whereas oxidative metabolism regenerates NAD^+^ from NADH in the complex I reaction (Birsoy et al., 2015; Sullivan et al., 2015). When cancer cells are starved of lipids, they must rely on *de novo* lipid synthesis to maintain cellular homeostasis. These cells compensate for this increased demand for lipid synthesis, and thus increased NAD^+^ requirement, by elevating ETC activity and their oxygen consumption to regenerate NAD^+^ (Li et al., 2022). Due to this demand for NAD^+^, lipid-starved cancer cells require oxygen and ETC activity to enable lipid synthesis and growth (Li et al., 2022). Thus, fluctuations in lipid availability indirectly alter ETC activity by modulating cellular NAD^+^ requirements, indicating that TME lipid availability affects mitochondrial oxidative metabolism in tumors.

In addition to affecting cellular metabolism by modulating substrates and cofactors available in cancer cells, lipid availability also affects cancer cell metabolism through nutrient-sensing pathways that detect lipid abundance in cells. For example, lipid availability regulates the HIF1α pathway and, thus, the cellular response to hypoxia. HIF1α target genes are upregulated when certain FABPs bind exogenous fatty acids (Seo et al., 2020). For instance, oleic acid stimulates the activity of FABP5, enhancing the translation of HIF1α. As HIF1α is critical for cancer cell adaptation to hypoxia, oleic acid might indirectly help cancer cells survive hypoxia by increasing HIF1α translation (Seo et al., 2020). Lipid availability in the TME also alters the way cancer cells metabolize glucose. Glucose transporter 1 (GLUT1, encoded by *SLC2A1*), which is responsible for glucose uptake at the cell membrane in cancer cells, requires *S*-palmitoylation to maintain membrane localization and glucose import (Zhang et al., 2021). *S*-palmitoylation is sensitive to levels of exogenous palmitate (Spinelli et al., 2018). Thus, glucose utilization by cancer cells might be regulated by palmitate availability in the TME. Interestingly, many nutrient transporters are *S*-palmitoylated (Villanueva and Hagenbuch, 2023), suggesting that the uptake of many nutrients in cancer cells is regulated by the availability of palmitate in the TME.

Lastly, various studies have shown that exogenous lipids significantly alter cellular metabolism via changes in histone modification (Eduardo et al., 2024 preprint; McDonnell et al., 2016; Yadav et al., 2022). In cells exposed to fatty acids, lipid oxidation leads to increased cellular levels of acetyl-CoA (McDonnell et al., 2016), which in turn leads to the increased acetylation of histones. As a result, exposure of a cell to fatty acids leads to significant changes in its chromatin packaging (McDonnell et al., 2016; Yadav et al., 2022). Thus, histone modifications driven by fatty acid exposure lead to altered gene expression, resulting in differential activity of many metabolic pathways. In particular, one-carbon metabolism (Box 1) and serine synthesis are both elevated in human breast cancer cell lines exposed to high levels of fatty acids *in vitro* (Eduardo et al., 2024 preprint; Yadav et al., 2022). Thus, lipids influence metabolism by acting as a source of acetyl-CoA for cancer cells.

These findings highlight intriguing links between lipid availability and diverse areas of tumor metabolism. Future studies are necessary to determine the tumor metabolic pathways affected by TME lipid availability and the mechanisms by which these lipids impact cancer cell metabolism.

## Conclusions and future directions

A consensus emerging from these studies is that the TME severely constrains the ability of cancer cells to synthesize lipids from canonical lipogenic pathways. Thus, tumors must rely on alternative lipid acquisition strategies. Another key conclusion is that lipid availability in the TME regulates other aspects of tumor metabolism. Lipid metabolism is highly interconnected with other metabolic pathways through shared cofactors, substrates and signaling pathways. Given this interconnection, TME lipid availability influences many metabolic properties of cancer cells, just as many non-lipid TME components influence lipid metabolism in cancer cells. To better understand the metabolic requirements of tumors, it is vitally important for future studies to: (1) accurately characterize the lipid composition of the TME, and (2) identify which TME lipids affect tumor biology and by what mechanisms.

Numerous emerging technologies will allow researchers to more accurately characterize lipids in the TME. Recent studies have isolated interstitial fluid from breast, colorectal, lung, melanoma, pancreatic and renal tumors to probe nutrient availability in the local microenvironment of these solid tumors (Apiz Saab and Muir, 2023). Lipidomic analysis can be applied to these interstitial fluid isolates to measure lipid availability in the TME (Altea-Manzano et al., 2023; Lien et al., 2021; Ringel et al., 2020; Xu et al., 2021; Zhang et al., 2017a). Rapidly maturing imaging mass spectrometry technologies will facilitate identification of the lipid species present in tumors and in specific regions and cell types (Alexandrov, 2023). Already, these imaging technologies are being applied to identify potential metabolic exchange between malignant and stromal cells in human lung cancer samples (Hu et al., 2023). Applying these techniques to investigate how lipid availability changes across tumor types, with disease progression and with therapeutic interventions will further enhance our understanding of which lipids are present in the TME.

Although emerging technologies are rapidly enabling the cataloging of lipids in the TME, the second goal – understanding the functional implications of TME lipid composition – remains challenging. New experimental tools are needed to answer these questions. Recently, novel systems, such as the fatty acid library for comprehensive ontologies (FALCON) system, have been developed to allow researchers to rapidly assess the functional effects of individual lipids on cellular phenotypes *ex vivo* (Wieder et al., 2023). Combined with information on the lipid composition of the TME, tools such as FALCON could identify TME lipids that functionally affect cancer cell biology. Additionally, the development of cell culture media with physiological levels of non-lipid nutrients has helped to identify previously unknown metabolic features of cancer (Ackermann and Tardito, 2019; Apiz Saab et al., 2023; Cantor, 2019; Cantor et al., 2017; Schug et al., 2015; Vande Voorde et al., 2019). The development of media with physiological levels of lipids could enable researchers to identify how lipids in the TME impact tumor metabolism, as well as the lipid species that contribute to these effects. This information will be crucial for identifying and targeting the metabolic requirements of cancers.

## References

[DMM050814C1] Ackerman, D., Tumanov, S., Qiu, B., Michalopoulou, E., Spata, M., Azzam, A., Xie, H., Simon, M. C. and Kamphorst, J. J. (2018). Triglycerides promote lipid homeostasis during hypoxic stress by balancing fatty acid saturation. *Cell Rep* 24, 2596-2605.e5. 10.1016/j.celrep.2018.08.015 30184495 PMC6137821

[DMM050814C2] Ackermann, T. and Tardito, S. (2019). Cell culture medium formulation and its implications in cancer metabolism. *Trends Cancer Res.* 5, 329-332. 10.1016/j.trecan.2019.05.004 PMC6557711 31208694

[DMM050814C3] Agarwala, P. K., Nie, S., Reid, G. E. and Kapoor, S. (2023). Global lipid remodelling by hypoxia aggravates migratory potential in pancreatic cancer while maintaining plasma membrane homeostasis. *Biochim. Biophys. Acta Mol. Cell Biol. Lipids* 1868, 159398. 10.1016/j.bbalip.2023.159398 37748704 PMC7616916

[DMM050814C4] Alexandrov, T. (2023). Spatial metabolomics: from a niche field towards a driver of innovation. *Nat Metab* 5, 1443-1445. 10.1038/s42255-023-00881-0 37679554

[DMM050814C5] Alicea, G. M., Rebecca, V. W., Goldman, A. R., Fane, M. E., Douglass, S. M., Behera, R., Webster, M. R., Kugel, C. H., Ecker, B. L., Cecilia Caino, M. et al. (2020). Changes in aged fibroblast lipid metabolism induce age-dependent melanoma cell resistance to targeted therapy via the fatty acid transporter FATP2. *Cancer Discov*. 10, 1282-1295. 10.1158/2159-8290.CD-20-0329 32499221 PMC7483379

[DMM050814C6] Altea-Manzano, P., Doglioni, G., Liu, Y., Cuadros, A. M., Nolan, E., Fernández-García, J., Wu, Q., Planque, M., Laue, K. J., Cidre-Aranaz, F. et al. (2023). A palmitate-rich metastatic niche enables metastasis growth via p65 acetylation resulting in pro-metastatic NF-κB signaling. *Nat. Cancer* 4, 344-364. 10.1038/s43018-023-00513-2 36732635 PMC7615234

[DMM050814C7] Apiz Saab, J. J. and Muir, A. (2023). Tumor interstitial fluid analysis enables the study of microenvironment-cell interactions in cancers. *Curr. Opin. Biotechnol.* 83, 102970. 10.1016/j.copbio.2023.102970 37494818 PMC10528471

[DMM050814C8] Apiz Saab, J. J., Dzierozynski, L. N., Jonker, P. B., AminiTabrizi, R., Shah, H., Menjivar, R. E., Scott, A. J., Nwosu, Z. C., Zhu, Z., Chen, R. N. et al. (2023). Pancreatic tumors exhibit myeloid-driven amino acid stress and upregulate arginine biosynthesis. *Elife* 12, e81289. 10.7554/eLife.81289 37254839 PMC10260022

[DMM050814C9] Auciello, F. R., Bulusu, V., Oon, C., Tait-Mulder, J., Berry, M., Bhattacharyya, S., Tumanov, S., Allen-Petersen, B. L., Link, J., Kendsersky, N. D. et al. (2019). A stromal lysolipid–autotaxin signaling axis promotes pancreatic tumor progression. *Cancer Discov.* 9, 617-627. 10.1158/2159-8290.CD-18-1212 30837243 PMC6497553

[DMM050814C10] Bensaad, K., Favaro, E., Lewis, C. A., Peck, B., Lord, S., Collins, J. M., Pinnick, K. E., Wigfield, S., Buffa, F. M., Li, J.-L. et al. (2014). Fatty acid uptake and lipid storage induced by HIF-1α contribute to cell growth and survival after hypoxia-reoxygenation. *Cell Rep.* 9, 349-365. 10.1016/j.celrep.2014.08.056 25263561

[DMM050814C11] Bielska, A. A., Harrigan, C. F., Kyung, Y. J., Morris, Q., Palm, W. and Thompson, C. B. (2022). Activating mTOR mutations are detrimental in nutrient-poor conditions. *Cancer Res.* 82, 3263-3274. 10.1158/0008-5472.CAN-22-0121 35857801 PMC10094744

[DMM050814C12] Birsoy, K., Wang, T., Chen, W. W., Freinkman, E., Abu-Remaileh, M. and Sabatini, D. M. (2015). An essential role of the mitochondrial electron transport chain in cell proliferation is to enable aspartate synthesis. *Cell* 162, 540-551. 10.1016/j.cell.2015.07.016 26232224 PMC4522279

[DMM050814C13] Blanquer-Rosselló, M. D. M., Oliver, J., Sastre-Serra, J., Valle, A. and Roca, P. (2016). Leptin regulates energy metabolism in MCF-7 breast cancer cells. *Int. J. Biochem. Cell Biol.* 72, 18-26. 10.1016/j.biocel.2016.01.002 26772821

[DMM050814C14] Broadfield, L. A., Pane, A. A., Talebi, A., Swinnen, J. V. and Fendt, S.-M. (2021). Lipid metabolism in cancer: New perspectives and emerging mechanisms. *Dev. Cell* 56, 1363-1393. 10.1016/j.devcel.2021.04.013 33945792

[DMM050814C15] Brown, M. S. and Goldstein, J. L. (1997). The SREBP pathway: regulation of cholesterol metabolism by proteolysis of a membrane-bound transcription factor. *Cell* 89, 331-340. 10.1016/S0092-8674(00)80213-5 9150132

[DMM050814C16] Cantor, J. R. (2019). The Rise of Physiologic Media. *Trends Cell Biol.* 29, 854-861. 10.1016/j.tcb.2019.08.009 31623927 PMC7001851

[DMM050814C17] Cantor, J. R., Abu-Remaileh, M., Kanarek, N., Freinkman, E., Gao, X., Louissaint, A., Jr, Lewis, C. A. and Sabatini, D. M. (2017). Physiologic medium rewires cellular metabolism and reveals uric acid as an endogenous inhibitor of UMP synthase. *Cell* 169, 258-272.e17. 10.1016/j.cell.2017.03.023 28388410 PMC5421364

[DMM050814C18] Caron, A., Richard, D. and Laplante, M. (2015). The roles of mTOR complexes in lipid metabolism. *Annu. Rev. Nutr.* 35, 321-348. 10.1146/annurev-nutr-071714-034355 26185979

[DMM050814C19] Challapalli, A., Carroll, L. and Aboagye, E. O. (2017). Molecular mechanisms of hypoxia in cancer. *Clin Transl Imaging* 5, 225-253. 10.1007/s40336-017-0231-1 28596947 PMC5437135

[DMM050814C20] Chandel, N. S. (2021). Lipid metabolism. *Cold Spring Harb. Perspect. Biol.* 13, a040576. 10.1101/cshperspect.a040576 34470787 PMC8411952

[DMM050814C21] Cheng, C., Ru, P., Geng, F., Liu, J., Yoo, J. Y., Wu, X., Cheng, X., Euthine, V., Hu, P., Guo, J. Y. et al. (2015). Glucose-mediated N-glycosylation of SCAP is essential for SREBP-1 activation and tumor growth. *Cancer Cell* 28, 569-581. 10.1016/j.ccell.2015.09.021 26555173 PMC4643405

[DMM050814C22] Cheng, M., Bhujwalla, Z. M. and Glunde, K. (2016). Targeting phospholipid metabolism in cancer. *Front. Oncol.* 6, 266. 10.3389/fonc.2016.00266 28083512 PMC5187387

[DMM050814C23] Cheng, C., Geng, F., Li, Z., Zhong, Y., Wang, H., Cheng, X., Zhao, Y., Mo, X., Horbinski, C., Duan, W. et al. (2022). Ammonia stimulates SCAP/Insig dissociation and SREBP-1 activation to promote lipogenesis and tumour growth. *Nat. Metab.* 4, 575-588. 10.1038/s42255-022-00568-y 35534729 PMC9177652

[DMM050814C24] Comerford, S. A., Huang, Z., Du, X., Wang, Y., Cai, L., Witkiewicz, A. K., Walters, H., Tantawy, M. N., Fu, A., Manning, H. C. et al. (2014). Acetate dependence of tumors. *Cell* 159, 1591-1602. 10.1016/j.cell.2014.11.020 25525877 PMC4272450

[DMM050814C25] Corbet, C. and Feron, O. (2017). Tumour acidosis: from the passenger to the driver's seat. *Nat. Rev. Cancer* 17, 577-593. 10.1038/nrc.2017.77 28912578

[DMM050814C26] Corbet, C., Pinto, A., Martherus, R., Santiago de Jesus, J. P., Polet, F. and Feron, O. (2016). Acidosis drives the reprogramming of fatty acid metabolism in cancer cells through changes in mitochondrial and histone acetylation. *Cell Metab.* 24, 311-323. 10.1016/j.cmet.2016.07.003 27508876

[DMM050814C27] Corbet, C., Bastien, E., Santiago de Jesus, J. P., Dierge, E., Martherus, R., Vander Linden, C., Doix, B., Degavre, C., Guilbaud, C., Petit, L. et al. (2020). TGFβ2-induced formation of lipid droplets supports acidosis-driven EMT and the metastatic spreading of cancer cells. *Nat. Commun.* 11, 1-15. 10.1038/s41467-019-14262-3 31974393 PMC6978517

[DMM050814C28] Corn, K. C., Windham, M. A. and Rafat, M. (2020). Lipids in the tumor microenvironment: From cancer progression to treatment. *Prog. Lipid Res.* 80, 101055. 10.1016/j.plipres.2020.101055 32791170 PMC7674189

[DMM050814C29] Dallmann, R., Viola, A. U., Tarokh, L., Cajochen, C. and Brown, S. A. (2012). The human circadian metabolome. *Proc. Natl. Acad. Sci. U. S. A* 109, 2625-2629. 10.1073/pnas.1114410109 22308371 PMC3289302

[DMM050814C30] Dang, C. V. and Semenza, G. L. (1999). Oncogenic alterations of metabolism. *Trends Biochem. Sci.* 24, 68-72. 10.1016/S0968-0004(98)01344-9 10098401

[DMM050814C31] de Candia, P., Prattichizzo, F., Garavelli, S., Alviggi, C., La Cava, A. and Matarese, G. (2021). The pleiotropic roles of leptin in metabolism, immunity, and cancer. *J. Exp. Med.* 218, e20191593. 10.1084/jem.20191593 33857282 PMC8056770

[DMM050814C32] de Visser, K. E. and Joyce, J. A. (2023). The evolving tumor microenvironment: From cancer initiation to metastatic outgrowth. *Cancer Cell* 41, 374-403. 10.1016/j.ccell.2023.02.016 36917948

[DMM050814C33] Deng, C.-F., Zhu, N., Zhao, T.-J., Li, H.-F., Gu, J., Liao, D.-F. and Qin, L. (2022). Involvement of LDL and ox-LDL in cancer development and its therapeutical potential. *Front. Oncol.* 12, 803473. 10.3389/fonc.2022.803473 35251975 PMC8889620

[DMM050814C34] Dickson, A. S., Pauzaite, T., Arnaiz, E., Ortmann, B. M., West, J. A., Volkmar, N., Martinelli, A. W., Li, Z., Wit, N., Vitkup, D. et al. (2023). A HIF independent oxygen-sensitive pathway for controlling cholesterol synthesis. *Nat. Commun.* 14, 4816. 10.1038/s41467-023-40541-1 37558666 PMC10412576

[DMM050814C35] Dierge, E., Debock, E., Guilbaud, C., Corbet, C., Mignolet, E., Mignard, L., Bastien, E., Dessy, C., Larondelle, Y. and Feron, O. (2021). Peroxidation of n-3 and n-6 polyunsaturated fatty acids in the acidic tumor environment leads to ferroptosis-mediated anticancer effects. *Cell Metab.* 33, 1701-1715.e5. 10.1016/j.cmet.2021.05.016 34118189

[DMM050814C36] Ding, M., Zhang, S., Guo, Y., Yao, J., Shen, Q., Huang, M., Chen, W., Yu, S., Zheng, Y., Lin, Y. et al. (2022). Tumor microenvironment acidity triggers lipid accumulation in liver cancer via SCD1 activation. *Mol. Cancer Res.* 20, 810-822. 10.1158/1541-7786.MCR-21-0699 35046108

[DMM050814C37] Dixon, S. J. and Olzmann, J. A. (2024). The cell biology of ferroptosis. *Nat. Rev. Mol. Cell Biol.* 25, 424-442. 10.1038/s41580-024-00703-5 38366038 PMC12187608

[DMM050814C38] Du, W., Zhang, L., Brett-Morris, A., Aguila, B., Kerner, J., Hoppel, C. L., Puchowicz, M., Serra, D., Herrero, L., Rini, B. I. et al. (2017). HIF drives lipid deposition and cancer in ccRCC via repression of fatty acid metabolism. *Nat. Commun.* 8, 1769. 10.1038/s41467-017-01965-8 29176561 PMC5701259

[DMM050814C39] Dudek, S. M. and Semenkovich, C. F. (1995). Essential amino acids regulate fatty acid synthase expression through an uncharged transfer RNA-dependent mechanism. *J. Biol. Chem.* 270, 29323-29329. 10.1074/jbc.270.49.29323 7493965

[DMM050814C40] DuFort, C. C., DelGiorno, K. E. and Hingorani, S. R. (2016). Mounting pressure in the microenvironment: fluids, solids, and cells in pancreatic ductal adenocarcinoma. *Gastroenterology* 150, 1545-1557.e2. 10.1053/j.gastro.2016.03.040 27072672 PMC4957812

[DMM050814C41] Eduardo, M. B., Cottone, G., McCloskey, C. W., Liu, S., Zappia, M. P., Benevolenskaya, E. V., Islam, A. B. M. M., Frolov, M. V., Palma, F. R., Gao, P. et al. (2024). Metabolic shift to serine pathway induced by lipids confers oncogenic properties in non-transformed breast cells. *bioRxiv* 2024.02.21.581404. 10.1101/2024.02.21.581404

[DMM050814C42] Efeyan, A., Comb, W. C. and Sabatini, D. M. (2015). Nutrient-sensing mechanisms and pathways. *Nature* 517, 302-310. 10.1038/nature14190 25592535 PMC4313349

[DMM050814C43] Elia, I. and Haigis, M. C. (2021). Metabolites and the tumour microenvironment: from cellular mechanisms to systemic metabolism. *Nat. Metab*. 3, 21-32. 10.1038/s42255-020-00317-z 33398194 PMC8097259

[DMM050814C44] El-Kenawi, A., Dominguez-Viqueira, W., Liu, M., Awasthi, S., Abraham-Miranda, J., Keske, A., Steiner, K. K., Noel, L., Serna, A. N., Dhillon, J. et al. (2021). Macrophage-derived cholesterol contributes to therapeutic resistance in prostate cancer. *Cancer Res.* 81, 5477-5490. 10.1158/0008-5472.CAN-20-4028 34301759 PMC8563406

[DMM050814C45] Fan, J., Kamphorst, J. J., Rabinowitz, J. D. and Shlomi, T. (2013). Fatty acid labeling from glutamine in hypoxia can be explained by isotope exchange without net reductive isocitrate dehydrogenase (IDH) flux. *J. Biol. Chem.* 288, 31363-31369. 10.1074/jbc.M113.502740 24030823 PMC3829450

[DMM050814C46] Faubert, B., Solmonson, A. and DeBerardinis, R. J. (2020). Metabolic reprogramming and cancer progression. *Science* 368, eaaw5473. 10.1126/science.aaw5473 32273439 PMC7227780

[DMM050814C47] Fendt, S.-M., Bell, E. L., Keibler, M. A., Olenchock, B. A., Mayers, J. R., Wasylenko, T. M., Vokes, N. I., Guarente, L., Vander Heiden, M. G. and Stephanopoulos, G. (2013). Reductive glutamine metabolism is a function of the α-ketoglutarate to citrate ratio in cells. *Nat. Commun.* 4, 2236. 10.1038/ncomms3236 23900562 PMC3934748

[DMM050814C48] Fendt, S.-M., Frezza, C. and Erez, A. (2020). Targeting metabolic plasticity and flexibility dynamics for cancer therapy. *Cancer Discov.* 10, 1797-1807. 10.1158/2159-8290.CD-20-0844 33139243 PMC7710573

[DMM050814C49] Ferraro, G. B., Ali, A., Luengo, A., Kodack, D. P., Deik, A., Abbott, K. L., Bezwada, D., Blanc, L., Prideaux, B., Jin, X. et al. (2021). Fatty acid synthesis is required for breast cancer brain metastasis. *Nat. Cancer* 2, 414-428. 10.1038/s43018-021-00183-y 34179825 PMC8223728

[DMM050814C50] Flowers, M. T. and Ntambi, J. M. (2008). Role of stearoyl-coenzyme A desaturase in regulating lipid metabolism. *Curr. Opin. Lipidol.* 19, 248-256. 10.1097/MOL.0b013e3282f9b54d 18460915 PMC4201499

[DMM050814C51] Fukumura, D., Duda, D. G., Munn, L. L. and Jain, R. K. (2010). Tumor microvasculature and microenvironment: Novel insights through intravital imaging in pre-clinical models. *Microcirculation* 17, 206-225. 10.1111/j.1549-8719.2010.00029.x 20374484 PMC2859831

[DMM050814C52] Gao, X., Lin, S.-H., Ren, F., Li, J.-T., Chen, J.-J., Yao, C.-B., Yang, H.-B., Jiang, S.-X., Yan, G.-Q., Wang, D. et al. (2016). Acetate functions as an epigenetic metabolite to promote lipid synthesis under hypoxia. *Nat. Commun.* 7, 11960. 10.1038/ncomms11960 27357947 PMC4931325

[DMM050814C53] Gao, X., Lee, K., Reid, M. A., Sanderson, S. M., Qiu, C., Li, S., Liu, J. and Locasale, J. W. (2018). Serine availability influences mitochondrial dynamics and function through lipid metabolism. *Cell Rep.* 22, 3507-3520. 10.1016/j.celrep.2018.03.017 29590619 PMC6054483

[DMM050814C54] Gharpure, K. M., Pradeep, S., Sans, M., Rupaimoole, R., Ivan, C., Wu, S. Y., Bayraktar, E., Nagaraja, A. S., Mangala, L. S., Zhang, X. et al. (2018). FABP4 as a key determinant of metastatic potential of ovarian cancer. *Nat. Commun.* 9, 2923. 10.1038/s41467-018-04987-y 30050129 PMC6062524

[DMM050814C55] Glatz, J. F. C., Luiken, J. J. F. P. and Bonen, A. (2010). Membrane fatty acid transporters as regulators of lipid metabolism: implications for metabolic disease. *Physiol. Rev.* 90, 367-417. 10.1152/physrev.00003.2009 20086080

[DMM050814C56] Goswami, S., Zhang, Q., Celik, C. E., Reich, E. M. and Yilmaz, Ö. H. (2023). Dietary fat and lipid metabolism in the tumor microenvironment. *Biochim. Biophys. Acta Rev. Cancer* 1878, 188984. 10.1016/j.bbcan.2023.188984 37722512 PMC10937091

[DMM050814C57] Green, C. R., Wallace, M., Divakaruni, A. S., Phillips, S. A., Murphy, A. N., Ciaraldi, T. P. and Metallo, C. M. (2016). Branched-chain amino acid catabolism fuels adipocyte differentiation and lipogenesis. *Nat. Chem. Biol.* 12, 15-21. 10.1038/nchembio.1961 26571352 PMC4684771

[DMM050814C58] Gullino, P. M., Grantham, F. H. and Smith, S. H. (1965). The interstitial water space of tumors. *Cancer Res.* 25, 727-731. 14347560

[DMM050814C59] Guo, F. and Cavener, D. R. (2007). The GCN2 eIF2α kinase regulates fatty-acid homeostasis in the liver during deprivation of an essential amino acid. *Cell Metab.* 5, 103-114. 10.1016/j.cmet.2007.01.001 17276353

[DMM050814C60] Hanahan, D. (2022). Hallmarks of Cancer: New Dimensions. *Cancer Discov.* 12, 31-46. 10.1158/2159-8290.CD-21-1059 35022204

[DMM050814C61] Harayama, T. and Riezman, H. (2018). Understanding the diversity of membrane lipid composition. *Nat. Rev. Mol. Cell Biol.* 19, 281-296. 10.1038/nrm.2017.138 29410529

[DMM050814C62] Hardie, D. G., Schaffer, B. E. and Brunet, A. (2016). AMPK: an energy-sensing pathway with multiple inputs and outputs. *Trends Cell Biol.* 26, 190-201. 10.1016/j.tcb.2015.10.013 26616193 PMC5881568

[DMM050814C63] Helmlinger, G., Sckell, A., Dellian, M., Forbes, N. S. and Jain, R. K. (2002). Acid production in glycolysis-impaired tumors provides new insights into tumor metabolism. *Clin. Cancer Res.* 8, 1284-1291. 11948144

[DMM050814C64] Herber, D. L., Cao, W., Nefedova, Y., Novitskiy, S. V., Nagaraj, S., Tyurin, V. A., Corzo, A., Cho, H.-I., Celis, E., Lennox, B. et al. (2010). Lipid accumulation and dendritic cell dysfunction in cancer. *Nat. Med.* 16, 880-886. 10.1038/nm.2172 20622859 PMC2917488

[DMM050814C65] Herzig, S. and Shaw, R. J. (2018). AMPK: guardian of metabolism and mitochondrial homeostasis. *Nat. Rev. Mol. Cell Biol.* 19, 121-135. 10.1038/nrm.2017.95 28974774 PMC5780224

[DMM050814C66] Ho, P.-C., Bihuniak, J. D., Macintyre, A. N., Staron, M., Liu, X., Amezquita, R., Tsui, Y.-C., Cui, G., Micevic, G., Perales, J. C. et al. (2015). Phosphoenolpyruvate Is a metabolic checkpoint of anti-tumor T cell responses. *Cell* 162, 1217-1228. 10.1016/j.cell.2015.08.012 26321681 PMC4567953

[DMM050814C67] Hollands, M. A. and Cawthorne, M. A. (1981). Important sites of lipogenesis in the mouse other than liver and white adipose tissue. *Biochem. J.* 196, 645-647. 10.1042/bj1960645 7317003 PMC1163042

[DMM050814C68] Hu, T., Allam, M., Cai, S., Henderson, W., Yueh, B., Garipcan, A., Ievlev, A. V., Afkarian, M., Beyaz, S. and Coskun, A. F. (2023). Single-cell spatial metabolomics with cell-type specific protein profiling for tissue systems biology. *Nat. Commun* 14, 8260. 10.1038/s41467-023-43917-5 38086839 PMC10716522

[DMM050814C69] Huang, D., Li, T., Li, X., Zhang, L., Sun, L., He, X., Zhong, X., Jia, D., Song, L., Semenza, G. L. et al. (2014). HIF-1-mediated suppression of acyl-CoA dehydrogenases and fatty acid oxidation is critical for cancer progression. *Cell Rep.* 8, 1930-1942. 10.1016/j.celrep.2014.08.028 25242319

[DMM050814C70] Iurlaro, R., León-Annicchiarico, C. L. and Muñoz-Pinedo, C. (2014). Chapter Three - regulation of cancer metabolism by oncogenes and tumor suppressors. In *Methods in Enzymology* (ed. L. Galluzzi and G. Kroemer), pp. 59-80. Academic Press. 10.1016/B978-0-12-416618-9.00003-0 24862260

[DMM050814C71] Jain, R. K. (2013). Normalizing tumor microenvironment to treat cancer: bench to bedside to biomarkers. *J. Clin. Oncol.* 31, 2205-2218. 10.1200/JCO.2012.46.3653 23669226 PMC3731977

[DMM050814C72] Jain, R. K., Martin, J. D. and Stylianopoulos, T. (2014). The role of mechanical forces in tumor growth and therapy. *Annu. Rev. Biomed. Eng.* 16, 321-346. 10.1146/annurev-bioeng-071813-105259 25014786 PMC4109025

[DMM050814C73] Jeon, S. M., Chandel, N. S. and Hay, N. (2012). AMPK regulates NADPH homeostasis to promote tumour cell survival during energy stress. *Nature* 485, 661-665. 10.1038/nature11066 22660331 PMC3607316

[DMM050814C74] Jiang, X., Stockwell, B. R. and Conrad, M. (2021). Ferroptosis: mechanisms, biology and role in disease. *Nat. Rev. Mol. Cell Biol.* 22, 266-282. 10.1038/s41580-020-00324-8 33495651 PMC8142022

[DMM050814C75] Jin, X., Demere, Z., Nair, K., Ali, A., Ferraro, G. B., Natoli, T., Deik, A., Petronio, L., Tang, A. A., Zhu, C. et al. (2020). A metastasis map of human cancer cell lines. *Nature* 588, 331-336. 10.1038/s41586-020-2969-2 33299191 PMC8439149

[DMM050814C76] Kamphorst, J. J., Cross, J. R., Fan, J., De Stanchina, E., Mathew, R., White, E. P., Thompson, C. B. and Rabinowitz, J. D. (2013). Hypoxic and Ras-transformed cells support growth by scavenging unsaturated fatty acids from lysophospholipids. *Proc. Natl. Acad. Sci. U. S. A* 110, 8882-8887. 10.1073/pnas.1307237110 23671091 PMC3670379

[DMM050814C77] Kaymak, I., Luda, K. M., Duimstra, L. R., Ma, E. H., Longo, J., Dahabieh, M. S., Faubert, B., Oswald, B. M., Watson, M. J., Kitchen-Goosen, S. M. et al. (2022). Carbon source availability drives nutrient utilization in CD8+ T cells. *Cell Metab.* 34, 1298-1311.e6. 10.1016/j.cmet.2022.07.012 35981545 PMC10068808

[DMM050814C78] Kondo, A., Yamamoto, S., Nakaki, R., Shimamura, T., Hamakubo, T., Sakai, J., Kodama, T., Yoshida, T., Aburatani, H. and Osawa, T. (2017). Extracellular acidic pH activates the sterol regulatory element-binding protein 2 to promote tumor progression. *Cell Rep.* 18, 2228-2242. 10.1016/j.celrep.2017.02.006 28249167

[DMM050814C79] Krishnan, J., Suter, M., Windak, R., Krebs, T., Felley, A., Montessuit, C., Tokarska-Schlattner, M., Aasum, E., Bogdanova, A., Perriard, E. et al. (2009). Activation of a HIF1alpha-PPARgamma axis underlies the integration of glycolytic and lipid anabolic pathways in pathologic cardiac hypertrophy. *Cell Metab.* 9, 512-524. 10.1016/j.cmet.2009.05.005 19490906

[DMM050814C80] Kumar, A., Cordes, T., Thalacker-Mercer, A. E., Pajor, A. M., Murphy, A. N. and Metallo, C. M. (2021). NaCT/SLC13A5 facilitates citrate import and metabolism under nutrient-limited conditions. *Cell Rep.* 36, 109701. 10.1016/j.celrep.2021.109701 34525352 PMC8500708

[DMM050814C81] Lamming, D. W. and Sabatini, D. M. (2013). A Central role for mTOR in lipid homeostasis. *Cell Metab.* 18, 465-469. 10.1016/j.cmet.2013.08.002 23973332 PMC3818790

[DMM050814C82] Lau, A. N. and Vander Heiden, M. G. (2020). Metabolism in the tumor microenvironment. *Ann. Rev. Cancer Biol.* 4, 17-40. 10.1146/annurev-cancerbio-030419-033333

[DMM050814C83] Lee, J. H., Cho, Y.-R., Kim, J. H., Kim, J., Nam, H. Y., Kim, S. W. and Son, J. (2019). Branched-chain amino acids sustain pancreatic cancer growth by regulating lipid metabolism. *Exp. Mol. Med.* 51, 1-11. 10.1038/s12276-019-0350-z PMC6884453 31784505

[DMM050814C84] Lewerenz, J., Hewett, S. J., Huang, Y., Lambros, M., Gout, P. W., Kalivas, P. W., Massie, A., Smolders, I., Methner, A., Pergande, M. et al. (2013). The cystine/glutamate antiporter system x(c)(-) in health and disease: from molecular mechanisms to novel therapeutic opportunities. *Antioxid. Redox Signal* 18, 522-555. 10.1089/ars.2011.4391 22667998 PMC3545354

[DMM050814C85] Li, Y., Su, X., Rohatgi, N., Zhang, Y., Brestoff, J. R., Shoghi, K. I., Xu, Y., Semenkovich, C. F., Harris, C. A., Peterson, L. L. et al. (2020). Hepatic lipids promote liver metastasis. *JCI Insight* 5, e136215. 10.1172/jci.insight.136215 32879136 PMC7487169

[DMM050814C86] Li, Z., Ji, B. W., Dixit, P. D., Tchourine, K., Lien, E. C., Hosios, A. M., Abbott, K. L., Rutter, J. C., Westermark, A. M., Gorodetsky, E. F. et al. (2022). Cancer cells depend on environmental lipids for proliferation when electron acceptors are limited. *Nat. Metab* 4, 711-723. 10.1038/s42255-022-00588-8 35739397 PMC10305743

[DMM050814C87] Liang, D., Minikes, A. M. and Jiang, X. (2022). Ferroptosis at the intersection of lipid metabolism and cellular signaling. *Mol. Cell* 82, 2215-2227. 10.1016/j.molcel.2022.03.022 35390277 PMC9233073

[DMM050814C88] Lien, E. C. and Vander Heiden, M. G. (2019). A framework for examining how diet impacts tumour metabolism. *Nat. Rev. Cancer* 19, 651-661. 10.1038/s41568-019-0198-5 31530936

[DMM050814C89] Lien, E. C., Westermark, A. M., Zhang, Y., Yuan, C., Li, Z., Lau, A. N., Sapp, K. M., Wolpin, B. M. and Vander Heiden, M. G. (2021). Low glycaemic diets alter lipid metabolism to influence tumour growth. *Nature* 599, 302-307. 10.1038/s41586-021-04049-2 34671163 PMC8628459

[DMM050814C90] Lim, A. R., Rathmell, W. K. and Rathmell, J. C. (2020). The tumor microenvironment as a metabolic barrier to effector T cells and immunotherapy. *Elife* 9, 1-13. 10.7554/eLife.55185 PMC7200151 32367803

[DMM050814C91] Lin, S.-C. and Hardie, D. G. (2018). AMPK: sensing glucose as well as cellular energy status. *Cell Metab.* 27, 299-313. 10.1016/j.cmet.2017.10.009 29153408

[DMM050814C92] Liu, J., Xu, A., Lam, K. S.-L., Wong, N.-S., Chen, J., Shepherd, P. R. and Wang, Y. (2013). Cholesterol-induced mammary tumorigenesis is enhanced by adiponectin deficiency: role of LDL receptor upregulation. *Oncotarget* 4, 1804-1818. 10.18632/oncotarget.1364 24113220 PMC3858565

[DMM050814C93] Liu, Q., Sun, Y., Fei, Z., Yang, Z., Duan, K., Zi, J., Cui, Q., Yu, M. and Xiong, W. (2019). Leptin promotes fatty acid oxidation and OXPHOS via the c-Myc/PGC-1 pathway in cancer cells. *Acta Biochim. Biophys. Sin.* 51, 707-714. 10.1093/abbs/gmz058 31187140

[DMM050814C94] Lone, M. A., Santos, T., Alecu, I., Silva, L. C. and Hornemann, T. (2019). 1-Deoxysphingolipids. *Biochim. Biophys. Acta Mol. Cell Biol. Lipids* 1864, 512-521. 10.1016/j.bbalip.2018.12.013 30625374

[DMM050814C95] Lopes-Coelho, F., André, S., Félix, A. and Serpa, J. (2018). Breast cancer metabolic cross-talk: Fibroblasts are hubs and breast cancer cells are gatherers of lipids. *Mol. Cell. Endocrinol.* 462, 93-106. 10.1016/j.mce.2017.01.031 28119133

[DMM050814C96] Lou, W., Gong, C., Ye, Z., Hu, Y., Zhu, M., Fang, Z. and Xu, H. (2022). Lipid metabolic features of T cells in the Tumor Microenvironment. *Lipids Health Dis.* 21, 94. 10.1186/s12944-022-01705-y 36203151 PMC9535888

[DMM050814C97] Lyssiotis, C. A. and Kimmelman, A. C. (2017). Metabolic interactions in the tumor microenvironment. *Trends Cell Biol.* 27, 863-875. 10.1016/j.tcb.2017.06.003 28734735 PMC5814137

[DMM050814C98] Ma, X., Xiao, L., Liu, L., Ye, L., Su, P., Bi, E., Wang, Q., Yang, M., Qian, J. and Yi, Q. (2021). CD36-mediated ferroptosis dampens intratumoral CD8+ T cell effector function and impairs their antitumor ability. *Cell Metab.* 33, 1001-1012.e5. 10.1016/j.cmet.2021.02.015 33691090 PMC8102368

[DMM050814C99] Magtanong, L., Ko, P.-J., To, M., Cao, J. Y., Forcina, G. C., Tarangelo, A. N., Ward, C. C., Cho, K. Y., Patti, G. J., Nomura, D. K. et al. (2019). Exogenous monounsaturated fatty acids promote a ferroptosis-resistant cell state. *Cell Chem. Biol.* 26, 420-432.e9. 10.1016/j.chembiol.2018.11.016 30686757 PMC6430697

[DMM050814C100] Mana, M. D., Hussey, A. M., Tzouanas, C. N., Imada, S., Barrera Millan, Y., Bahceci, D., Saiz, D. R., Webb, A. T., Lewis, C. A., Carmeliet, P. et al. (2021). High-fat diet-activated fatty acid oxidation mediates intestinal stemness and tumorigenicity. *Cell Rep* 35, 109212. 10.1016/j.celrep.2021.109212 34107251 PMC8258630

[DMM050814C101] Marelli, G., Morina, N., Portale, F., Pandini, M., Iovino, M., Di Conza, G., Ho, P.-C. and Di Mitri, D. (2022). Lipid-loaded macrophages as new therapeutic target in cancer. *J. Immunother Cancer* 10, e004584. 10.1136/jitc-2022-004584 35798535 PMC9263925

[DMM050814C102] Martínez-Reyes, I. and Chandel, N. S. (2021). Cancer metabolism: looking forward. *Nat. Rev. Cancer* 21, 669-680. 10.1038/s41568-021-00378-6 34272515

[DMM050814C103] Masetti, M., Carriero, R., Portale, F., Marelli, G., Morina, N., Pandini, M., Iovino, M., Partini, B., Erreni, M., Ponzetta, A. et al. (2022). Lipid-loaded tumor-associated macrophages sustain tumor growth and invasiveness in prostate cancer. *J. Exp. Med.* 219, e20210564. 10.1084/jem.20210564 34919143 PMC8932635

[DMM050814C104] McDonnell, E., Crown, S. B., Fox, D. B., Kitir, B., Ilkayeva, O. R., Olsen, C. A., Grimsrud, P. A. and Hirschey, M. D. (2016). Lipids reprogram metabolism to become a major carbon source for histone acetylation. *Cell Rep.* 17, 1463-1472. 10.1016/j.celrep.2016.10.012 27806287 PMC5123807

[DMM050814C105] Menard, J. A., Christianson, H. C., Kucharzewska, P., Bourseau-Guilmain, E., Svensson, K. J., Lindqvist, E., Indira Chandran, V., Kjellén, L., Welinder, C., Bengzon, J. et al. (2016). Metastasis stimulation by hypoxia and acidosis-induced extracellular lipid uptake is mediated by proteoglycan-dependent endocytosis. *Cancer Res.* 76, 4828-4840. 10.1158/0008-5472.CAN-15-2831 27199348

[DMM050814C106] Menendez, J. A. and Lupu, R. (2007). Fatty acid synthase and the lipogenic phenotype in cancer pathogenesis. *Nat. Rev. Cancer* 7, 763-777. 10.1038/nrc2222 17882277

[DMM050814C107] Menjivar, R. E., Nwosu, Z. C., Du, W., Donahue, K. L., Hong, H. S., Espinoza, C., Brown, K., Velez-Delgado, A., Yan, W., Lima, F. et al. (2023). Arginase 1 is a key driver of immune suppression in pancreatic cancer. *Elife* 12, e80721. 10.7554/eLife.80721 36727849 PMC10260021

[DMM050814C108] Metallo, C. M., Gameiro, P. A., Bell, E. L., Mattaini, K. R., Yang, J., Hiller, K., Jewell, C. M., Johnson, Z. R., Irvine, D. J., Guarente, L. et al. (2011). Reductive glutamine metabolism by IDH1 mediates lipogenesis under hypoxia. *Nature* 481, 380-384. 10.1038/nature10602 22101433 PMC3710581

[DMM050814C109] Miess, H., Dankworth, B., Gouw, A. M., Rosenfeldt, M., Schmitz, W., Jiang, M., Saunders, B., Howell, M., Downward, J., Felsher, D. W. et al. (2018). The glutathione redox system is essential to prevent ferroptosis caused by impaired lipid metabolism in clear cell renal cell carcinoma. *Oncogene* 37, 5435-5450. 10.1038/s41388-018-0315-z 29872221 PMC6173300

[DMM050814C110] Miska, J., Lee-Chang, C., Rashidi, A., Muroski, M. E., Chang, A. L., Lopez-Rosas, A., Zhang, P., Panek, W. K., Cordero, A., Han, Y. et al. (2022). HIF-1α is a metabolic switch between glycolytic-driven migration and oxidative phosphorylation-driven immunosuppression of Tregs in glioblastoma. *Cell Rep.* 39, 110934. 10.1016/j.celrep.2022.110934 35675772

[DMM050814C111] Muir, A. and Vander Heiden, M. G. (2018). The nutrient environment affects therapy. *Science* 360, 962-963. 10.1126/science.aar5986 29853672 PMC6368963

[DMM050814C112] Muir, A., Danai, L. V. and Vander Heiden, M. G. (2018). Microenvironmental regulation of cancer cell metabolism: implications for experimental design and translational studies. *Dis. Model. Mech.* 11, dmm035758. 10.1242/dmm.035758 30104199 PMC6124553

[DMM050814C113] Mukherjee, A., Chiang, C.-Y., Daifotis, H. A., Nieman, K. M., Fahrmann, J. F., Lastra, R. R., Romero, I. L., Fiehn, O. and Lengyel, E. (2020). Adipocyte-induced FABP4 expression in ovarian cancer cells promotes metastasis and mediates carboplatin resistance. *Cancer Res.* 80, 1748-1761. 10.1158/0008-5472.CAN-19-1999 32054768 PMC10656748

[DMM050814C114] Mukherjee, A., Bezwada, D., Greco, F., Zandbergen, M., Shen, T., Chiang, C.-Y., Tasdemir, M., Fahrmann, J., Grapov, D., La Frano, M. R. et al. (2023). Adipocytes reprogram cancer cell metabolism by diverting glucose towards glycerol-3-phosphate thereby promoting metastasis. *Nat. Metabolism* 5, 1563-1577. 10.1038/s42255-023-00879-8 PMC12175003 37653041

[DMM050814C115] Mullen, A. R., Wheaton, W. W., Jin, E. S., Chen, P.-H., Sullivan, L. B., Cheng, T., Yang, Y., Linehan, W. M., Chandel, N. S. and DeBerardinis, R. J. (2011). Reductive carboxylation supports growth in tumour cells with defective mitochondria. *Nature* 481, 385-388. 10.1038/nature10642 22101431 PMC3262117

[DMM050814C116] Murthy, D., Attri, K. S., Shukla, S. K., Thakur, R., Chaika, N. V., He, C., Wang, D., Jha, K., Dasgupta, A., King, R. J. et al. (2024). Cancer-associated fibroblast-derived acetate promotes pancreatic cancer development by altering polyamine metabolism via the ACSS2-SP1-SAT1 axis. *Nat. Cell Biol.* 26, 613-627. 10.1038/s41556-024-01372-4 38429478 PMC11021164

[DMM050814C117] Muthusamy, T., Cordes, T., Handzlik, M. K., You, L., Lim, E. W., Gengatharan, J., Pinto, A. F. M., Badur, M. G., Kolar, M. J., Wallace, M. et al. (2020). Serine restriction alters sphingolipid diversity to constrain tumour growth. *Nature* 586, 790-795. 10.1038/s41586-020-2609-x 32788725 PMC7606299

[DMM050814C118] Nagarajan, A., Malvi, P. and Wajapeyee, N. (2016). Oncogene-directed alterations in cancer cell metabolism. *Trends Cancer Res.* 2, 365-377. 10.1016/j.trecan.2016.06.002 PMC5096652 27822561

[DMM050814C119] Nakahara, R., Aki, S., Sugaya, M., Hirose, H., Kato, M., Maeda, K., Sakamoto, D. M., Kojima, Y., Nishida, M., Ando, R. et al. (2023). Hypoxia activates SREBP2 through Golgi disassembly in bone marrow-derived monocytes for enhanced tumor growth. *EMBO J.* 42, e114032. 10.15252/embj.2023114032 37781951 PMC10646561

[DMM050814C120] Nardi, F., Fitchev, P., Brooks, K. M., Franco, O. E., Cheng, K., Hayward, S. W., Welte, M. A. and Crawford, S. E. (2019). Lipid droplet velocity is a microenvironmental sensor of aggressive tumors regulated by V-ATPase and PEDF. *Lab. Invest* 99, 1822-1834. 10.1038/s41374-019-0296-8 31409893 PMC7289525

[DMM050814C121] Nguyen, T. B., Louie, S. M., Daniele, J. R., Tran, Q., Dillin, A., Zoncu, R., Nomura, D. K. and Olzmann, J. A. (2017). DGAT1-dependent lipid droplet biogenesis protects mitochondrial function during starvation-induced autophagy. *Dev. Cell* 42, 9-21.e5. 10.1016/j.devcel.2017.06.003 28697336 PMC5553613

[DMM050814C122] Nieman, K. M., Kenny, H. A., Penicka, C. V., Ladanyi, A., Buell-Gutbrod, R., Zillhardt, M. R., Romero, I. L., Carey, M. S., Mills, G. B., Hotamisligil, G. S. et al. (2011). Adipocytes promote ovarian cancer metastasis and provide energy for rapid tumor growth. *Nat. Med.* 17, 1498-1503. 10.1038/nm.2492 22037646 PMC4157349

[DMM050814C123] Nwosu, Z. C., Ward, M. H., Sajjakulnukit, P., Poudel, P., Ragulan, C., Kasperek, S., Radyk, M., Sutton, D., Menjivar, R. E., Andren, A. et al. (2023). Uridine-derived ribose fuels glucose-restricted pancreatic cancer. *Nature* 618, 151-158. 10.1038/s41586-023-06073-w 37198494 PMC10232363

[DMM050814C124] Ogretmen, B. (2018). Sphingolipid metabolism in cancer signalling and therapy. *Nat. Rev. Cancer* 18, 33-50. 10.1038/nrc.2017.96 29147025 PMC5818153

[DMM050814C125] Olive, K. P., Jacobetz, M. A., Davidson, C. J., Gopinathan, A., McIntyre, D., Honess, D., Madhu, B., Goldgraben, M. A., Caldwell, M. E., Allard, D. et al. (2009). Inhibition of Hedgehog signaling enhances delivery of chemotherapy in a mouse model of pancreatic cancer. *Science* 324, 1457-1461. 10.1126/science.1171362 19460966 PMC2998180

[DMM050814C126] Olzmann, J. A. and Carvalho, P. (2019). Dynamics and functions of lipid droplets. *Nat. Rev. Mol. Cell Biol.* 20, 137-155. 10.1038/s41580-018-0085-z 30523332 PMC6746329

[DMM050814C127] Palm, W. and Thompson, C. B. (2017). Nutrient acquisition strategies of mammalian cells. *Nature* 546, 234-242. 10.1038/nature22379 28593971 PMC5541675

[DMM050814C128] Palm, W., Park, Y., Wright, K., Pavlova, N. N., Tuveson, D. A. and Thompson, C. B. (2015). The Utilization of Extracellular Proteins as Nutrients Is Suppressed by mTORC1. *Cell* 162, 259-270. 10.1016/j.cell.2015.06.017 26144316 PMC4506698

[DMM050814C129] Pascual, G., Avgustinova, A., Mejetta, S., Martín, M., Castellanos, A., Attolini, C. S.-O., Berenguer, A., Prats, N., Toll, A., Hueto, J. A. et al. (2017). Targeting metastasis-initiating cells through the fatty acid receptor CD36. *Nature* 541, 41-45. 10.1038/nature20791 27974793

[DMM050814C130] Paton, C. M. and Ntambi, J. M. (2009). Biochemical and physiological function of stearoyl-CoA desaturase. *Am. J. Physiol. Endocrinol. Metab.* 297, E28-E37. 10.1152/ajpendo.90897.2008 19066317 PMC2711665

[DMM050814C131] Pham, D.-V., Tilija Pun, N. and Park, P.-H. (2021). Autophagy activation and SREBP-1 induction contribute to fatty acid metabolic reprogramming by leptin in breast cancer cells. *Mol. Oncol.* 15, 657-678. 10.1002/1878-0261.12860 33226729 PMC7858107

[DMM050814C132] Prendeville, H. and Lynch, L. (2022). Diet, lipids, and antitumor immunity. *Cell. Mol. Immunol.* 19, 432-444. 10.1038/s41423-021-00781-x 34983949 PMC8891265

[DMM050814C133] Qiu, B., Ackerman, D., Sanchez, D. J., Li, B., Ochocki, J. D., Grazioli, A., Bobrovnikova-Marjon, E., Diehl, J. A., Keith, B. and Simon, M. C. (2015). HIF2α-dependent lipid storage promotes endoplasmic reticulum homeostasis in clear-cell renal cell carcinoma. *Cancer Discov.* 5, 652-667. 10.1158/2159-8290.CD-14-1507 25829424 PMC4456212

[DMM050814C134] Quehenberger, O. and Dennis, E. A. (2011). The human plasma lipidome. *N. Engl. J. Med.* 365, 1812-1823. 10.1056/NEJMra1104901 22070478 PMC3412394

[DMM050814C135] Quehenberger, O., Armando, A. M., Brown, A. H., Milne, S. B., Myers, D. S., Merrill, A. H., Bandyopadhyay, S., Jones, K. N., Kelly, S., Shaner, R. L. et al. (2010). Lipidomics reveals a remarkable diversity of lipids in human plasma. *J. Lipid Res.* 51, 3299-3305. 10.1194/jlr.M009449 20671299 PMC2952570

[DMM050814C136] Ricoult, S. J. H. and Manning, B. D. (2013). The multifaceted role of mTORC1 in the control of lipid metabolism. *EMBO Rep.* 14, 242-251. 10.1038/embor.2013.5 23399656 PMC3589096

[DMM050814C137] Ringel, A. E., Drijvers, J. M., Baker, G. J., Catozzi, A., García-Cañaveras, J. C., Gassaway, B. M., Miller, B. C., Juneja, V. R., Nguyen, T. H., Joshi, S. et al. (2020). Obesity shapes metabolism in the tumor microenvironment to suppress anti-tumor immunity. *Cell* 183, 1848-1866.e26. 10.1016/j.cell.2020.11.009 33301708 PMC8064125

[DMM050814C138] Röhrig, F. and Schulze, A. (2016). The multifaceted roles of fatty acid synthesis in cancer. *Nat. Rev. Cancer* 16, 732-749. 10.1038/nrc.2016.89 27658529

[DMM050814C139] Salloum, G., Bresnick, A. R. and Backer, J. M. (2023). Macropinocytosis: mechanisms and regulation. *Biochem. J.* 480, 335-362. 10.1042/BCJ20210584 36920093

[DMM050814C140] Schug, Z. T., Peck, B., Jones, D. T., Zhang, Q., Grosskurth, S., Alam, I. S., Goodwin, L. M., Smethurst, E., Mason, S., Blyth, K. et al. (2015). Acetyl-CoA synthetase 2 promotes acetate utilization and maintains cancer cell growth under metabolic stress. *Cancer Cell* 27, 57-71. 10.1016/j.ccell.2014.12.002 25584894 PMC4297291

[DMM050814C141] Sen, U., Coleman, C. and Sen, T. (2023). Stearoyl coenzyme A desaturase-1: multitasker in cancer, metabolism, and ferroptosis. *Trends Cancer Res.* 9, 480-489. 10.1016/j.trecan.2023.03.003 37029018

[DMM050814C142] Seo, J., Jeong, D. W., Park, J. W., Lee, K. W., Fukuda, J. and Chun, Y. S. (2020). Fatty-acid-induced FABP5/HIF-1 reprograms lipid metabolism and enhances the proliferation of liver cancer cells. *Commun. Biol.* 3, 638. 10.1038/s42003-020-01367-5 33128030 PMC7599230

[DMM050814C143] Shen, G.-M., Zhao, Y.-Z., Chen, M.-T., Zhang, F.-L., Liu, X.-L., Wang, Y., Liu, C.-Z., Yu, J. and Zhang, J.-W. (2012). Hypoxia-inducible factor-1 (HIF-1) promotes LDL and VLDL uptake through inducing VLDLR under hypoxia. *Biochem. J.* 441, 675-683. 10.1042/BJ20111377 21970364

[DMM050814C144] Shimano, H. and Sato, R. (2017). SREBP-regulated lipid metabolism: convergent physiology — divergent pathophysiology. *Nat. Rev. Endocrinol.* 13, 710-730. 10.1038/nrendo.2017.91 28849786

[DMM050814C145] Snaebjornsson, M. T., Janaki-Raman, S. and Schulze, A. (2020). Greasing the wheels of the cancer machine: the role of lipid metabolism in cancer. *Cell Metab.* 31, 62-76. 10.1016/j.cmet.2019.11.010 31813823

[DMM050814C146] Sousa, C. M., Biancur, D. E., Wang, X., Halbrook, C. J., Sherman, M. H., Zhang, L., Kremer, D., Hwang, R. F., Witkiewicz, A. K., Ying, H. et al. (2016). Pancreatic stellate cells support tumour metabolism through autophagic alanine secretion. *Nature* 536, 479-483. 10.1038/nature19084 27509858 PMC5228623

[DMM050814C147] Spinelli, J. B., Yoon, H., Ringel, A. E., Jeanfavre, S., Clish, C. B. and Haigis, M. C. (2017). Metabolic recycling of ammonia via glutamate dehydrogenase supports breast cancer biomass. *Science* 358, 941-946. 10.1126/science.aam9305 29025995 PMC5748897

[DMM050814C148] Spinelli, M., Fusco, S. and Grassi, C. (2018). Nutrient-dependent changes of protein palmitoylation: impact on nuclear enzymes and regulation of gene expression. *Int. J. Mol. Sci.* 19, 3820. 10.3390/ijms19123820 30513609 PMC6320809

[DMM050814C149] Stine, Z. E., Schug, Z. T., Salvino, J. M. and Dang, C. V. (2022). Targeting cancer metabolism in the era of precision oncology. *Nat. Rev. Drug Discov.* 21, 141-162. 10.1038/s41573-021-00339-6 34862480 PMC8641543

[DMM050814C150] Su, P., Wang, Q., Bi, E., Ma, X., Liu, L., Yang, M., Qian, J. and Yi, Q. (2020). Enhanced lipid accumulation and metabolism are required for the differentiation and activation of tumor-associated macrophages. *Cancer Res.* 80, 1438-1450. 10.1158/0008-5472.CAN-19-2994 32015091 PMC7127942

[DMM050814C151] Sullivan, L. B., Gui, D. Y., Hosios, A. M., Bush, L. N., Freinkman, E. and Vander Heiden, M. G. (2015). Supporting aspartate biosynthesis is an essential function of respiration in proliferating cells. *Cell* 162, 552-563. 10.1016/j.cell.2015.07.017 26232225 PMC4522278

[DMM050814C152] Sun, R. C. and Denko, N. C. (2014). Hypoxic regulation of glutamine metabolism through HIF1 and SIAH2 supports lipid synthesis that is necessary for tumor growth. *Cell Metab.* 19, 285-292. 10.1016/j.cmet.2013.11.022 24506869 PMC3920584

[DMM050814C153] Sunshine, H. and Iruela-Arispe, M. L. (2017). Membrane lipids and cell signaling. *Curr. Opin. Lipidol* 28, 408-413. 10.1097/MOL.0000000000000443 28692598 PMC5776726

[DMM050814C154] Swietach, P., Boedtkjer, E. and Pedersen, S. F. (2023). How protons pave the way to aggressive cancers. *Nat. Rev. Cancer* 23, 825-841. 10.1038/s41568-023-00628-9 37884609

[DMM050814C155] Tirinato, L., Pagliari, F., Limongi, T., Marini, M., Falqui, A., Seco, J., Candeloro, P., Liberale, C. and Di Fabrizio, E. (2017). An overview of lipid droplets in cancer and cancer stem cells. *Stem Cells Int.* 2017, 1656053. 10.1155/2017/1656053 28883835 PMC5572636

[DMM050814C156] Torrence, M. E. and Manning, B. D. (2018). Nutrient sensing in cancer. *Annu. Rev. Cancer Biol.* 2, 251-269. 10.1146/annurev-cancerbio-030617-050329

[DMM050814C157] Ubellacker, J. M., Tasdogan, A., Ramesh, V., Shen, B., Mitchell, E. C., Martin-Sandoval, M. S., Gu, Z., McCormick, M. L., Durham, A. B., Spitz, D. R. et al. (2020). Lymph protects metastasizing melanoma cells from ferroptosis. *Nature* 585, 113-118. 10.1038/s41586-020-2623-z 32814895 PMC7484468

[DMM050814C158] Van der Horst, D. J., Roosendaal, S. D. and Rodenburg, K. W. (2009). Circulatory lipid transport: lipoprotein assembly and function from an evolutionary perspective. *Mol. Cell. Biochem.* 326, 105-119. 10.1007/s11010-008-0011-3 19130182

[DMM050814C159] Vande Voorde, J., Ackermann, T., Pfetzer, N., Sumpton, D., Mackay, G., Kalna, G., Nixon, C., Blyth, K., Gottlieb, E. and Tardito, S. (2019). Improving the metabolic fidelity of cancer models with a physiological cell culture medium. *Sci. Adv.* 5, eaau7314. 10.1126/sciadv.aau7314 30613774 PMC6314821

[DMM050814C160] VandeKopple, M. J., Wu, J., Auer, E. N., Giaccia, A. J., Denko, N. C. and Papandreou, I. (2019). HILPDA regulates lipid metabolism, lipid droplet abundance, and response to microenvironmental stress in solid tumors. *Mol. Cancer Res.* 17, 2089-2101. 10.1158/1541-7786.MCR-18-1343 31308147 PMC6774878

[DMM050814C161] Vander Heiden, M. G. and DeBerardinis, R. J. (2017). Understanding the intersections between metabolism and cancer biology. *Cell* 168, 657-669. 10.1016/j.cell.2016.12.039 28187287 PMC5329766

[DMM050814C162] Villanueva, C. E. and Hagenbuch, B. (2023). Palmitoylation of solute carriers. *Biochem. Pharmacol.* 215, 115695. 10.1016/j.bcp.2023.115695 37481134 PMC10530500

[DMM050814C163] Vitale, I., Manic, G., Coussens, L. M., Kroemer, G. and Galluzzi, L. (2019). Macrophages and metabolism in the tumor microenvironment. *Cell Metab.* 30, 36-50. 10.1016/j.cmet.2019.06.001 31269428

[DMM050814C164] Vogel, F. C. E., Chaves-Filho, A. B. and Schulze, A. (2024). Lipids as mediators of cancer progression and metastasis. *Nat. Cancer* 5, 16-29. 10.1038/s43018-023-00702-z 38273023

[DMM050814C165] Volmer, R., van der Ploeg, K. and Ron, D. (2013). Membrane lipid saturation activates endoplasmic reticulum unfolded protein response transducers through their transmembrane domains. *Proc. Natl. Acad. Sci. USA* 110, 4628-4633. 10.1073/pnas.1217611110 23487760 PMC3606975

[DMM050814C166] Walther, T. C. and Farese, R. V., Jr (2012). Lipid droplets and cellular lipid metabolism. *Annu. Rev. Biochem.* 81, 687-714. 10.1146/annurev-biochem-061009-102430 22524315 PMC3767414

[DMM050814C167] Wang, Y. Y., Attané, C., Milhas, D., Dirat, B., Dauvillier, S., Guerard, A., Gilhodes, J., Lazar, I., Alet, N., Laurent, V. et al. (2017). Mammary adipocytes stimulate breast cancer invasion through metabolic remodeling of tumor cells. *JCI Insight* 2, e87489. 10.1172/jci.insight.87489 28239646 PMC5313068

[DMM050814C168] Wang, T., Fahrmann, J. F., Lee, H., Li, Y.-J., Tripathi, S. C., Yue, C., Zhang, C., Lifshitz, V., Song, J., Yuan, Y. et al. (2018). JAK/STAT3-regulated fatty acid β-oxidation is critical for breast cancer stem cell self-renewal and chemoresistance. *Cell Metab.* 27, 136-150.e5. 10.1016/j.cmet.2017.11.001 29249690 PMC5777338

[DMM050814C169] Wang, W., Green, M., Choi, J. E., Gijón, M., Kennedy, P. D., Johnson, J. K., Liao, P., Lang, X., Kryczek, I., Sell, A. et al. (2019). CD8+ T cells regulate tumour ferroptosis during cancer immunotherapy. *Nature* 569, 270-274. 10.1038/s41586-019-1170-y 31043744 PMC6533917

[DMM050814C170] Wang, H., Franco, F., Tsui, Y.-C., Xie, X., Trefny, M. P., Zappasodi, R., Mohmood, S. R., Fernández-García, J., Tsai, C.-H., Schulze, I. et al. (2020). CD36-mediated metabolic adaptation supports regulatory T cell survival and function in tumors. *Nat. Immunol.* 21, 298-308. 10.1038/s41590-019-0589-5 32066953 PMC7043937

[DMM050814C171] Webb, B. A., Chimenti, M., Jacobson, M. P. and Barber, D. L. (2011). Dysregulated pH: a perfect storm for cancer progression. *Nat. Rev. Cancer* 11, 671-677. 10.1038/nrc3110 21833026

[DMM050814C172] Wieder, N., Fried, J. C., Kim, C., Sidhom, E.-H., Brown, M. R., Marshall, J. L., Arevalo, C., Dvela-Levitt, M., Kost-Alimova, M., Sieber, J. et al. (2023). FALCON systematically interrogates free fatty acid biology and identifies a novel mediator of lipotoxicity. *Cell Metab.* 35, 887-905.e11. 10.1016/j.cmet.2023.03.018 37075753 PMC10257950

[DMM050814C173] Wilson, W. R. and Hay, M. P. (2011). Targeting hypoxia in cancer therapy. *Nat. Rev. Cancer* 11, 393-410. 10.1038/nrc3064 21606941

[DMM050814C174] Wise, D. R., Ward, P. S., Shay, J. E. S., Cross, J. R., Gruber, J. J., Sachdeva, U. M., Platt, J. M., DeMatteo, R. G., Simon, M. C. and Thompson, C. B. (2011). Hypoxia promotes isocitrate dehydrogenase-dependent carboxylation of α-ketoglutarate to citrate to support cell growth and viability. *Proc. Natl. Acad. Sci. U. S. A* 108, 19611-19616. 10.1073/pnas.1117773108 22106302 PMC3241793

[DMM050814C175] Xiao, F., Wang, C., Yin, H., Yu, J., Chen, S., Fang, J. and Guo, F. (2016). Leucine deprivation inhibits proliferation and induces apoptosis of human breast cancer cells via fatty acid synthase. *Oncotarget* 7, 63679-63689. 10.18632/oncotarget.11626 27579768 PMC5325395

[DMM050814C176] Xu, S., Chaudhary, O., Rodríguez-Morales, P., Sun, X., Chen, D., Zappasodi, R., Xu, Z., Pinto, A. F. M., Williams, A., Schulze, I. et al. (2021). Uptake of oxidized lipids by the scavenger receptor CD36 promotes lipid peroxidation and dysfunction in CD8+ T cells in tumors. *Immunity* 54, 1561-1577.e7. 10.1016/j.immuni.2021.05.003 34102100 PMC9273026

[DMM050814C177] Yadav, S., Virk, R., Chung, C. H., Eduardo, M. B., VanDerway, D., Chen, D., Burdett, K., Gao, H., Zeng, Z., Ranjan, M. et al. (2022). Lipid exposure activates gene expression changes associated with estrogen receptor negative breast cancer. *NPJ Breast Cancer* 8, 59. 10.1038/s41523-022-00422-0 35508495 PMC9068822

[DMM050814C178] Yoon, H., Shaw, J. L., Haigis, M. C. and Greka, A. (2021). Lipid metabolism in sickness and in health: Emerging regulators of lipotoxicity. *Mol. Cell* 81, 3708-3730. 10.1016/j.molcel.2021.08.027 34547235 PMC8620413

[DMM050814C179] Young, R. M., Ackerman, D., Quinn, Z. L., Mancuso, A., Gruber, M., Liu, L., Giannoukos, D. N., Bobrovnikova-Marjon, E., Diehl, J. A., Keith, B. et al. (2013). Dysregulated mTORC1 renders cells critically dependent on desaturated lipids for survival under tumor-like stress. *Genes Dev.* 27, 1115-1131. 10.1101/gad.198630.112 23699409 PMC3672646

[DMM050814C180] Zadoorian, A., Du, X. and Yang, H. (2023). Lipid droplet biogenesis and functions in health and disease. *Nat. Rev. Endocrinol.* 19, 443-459. 10.1038/s41574-023-00845-0 37221402 PMC10204695

[DMM050814C181] Zhang, Y., Kurupati, R., Liu, L., Zhou, X. Y., Zhang, G., Hudaihed, A., Filisio, F., Giles-Davis, W., Xu, X., Karakousis, G. C. et al. (2017a). Enhancing CD8+ T cell fatty acid catabolism within a metabolically challenging tumor microenvironment increases the efficacy of melanoma immunotherapy. *Cancer Cell* 32, 377-391.e9. 10.1016/j.ccell.2017.08.004 28898698 PMC5751418

[DMM050814C182] Zhang, X., Saarinen, A. M., Hitosugi, T., Wang, Z., Wang, L., Ho, T. H. and Liu, J. (2017b). Inhibition of intracellular lipolysis promotes human cancer cell adaptation to hypoxia. *Elife* 6, e31132. 10.7554/eLife.31132 29256392 PMC5739538

[DMM050814C183] Zhang, M., Di Martino, J. S., Bowman, R. L., Campbell, N. R., Baksh, S. C., Simon-Vermot, T., Kim, I. S., Haldeman, P., Mondal, C., Yong-Gonzales, V. et al. (2018). Adipocyte-derived lipids mediate melanoma progression via FATP proteins. *Cancer Discov.* 8, 1006-1025. 10.1158/2159-8290.CD-17-1371 29903879 PMC6192670

[DMM050814C184] Zhang, Z., Li, X., Yang, F., Chen, C., Liu, P., Ren, Y., Sun, P., Wang, Z., You, Y., Zeng, Y.-X. et al. (2021). DHHC9-mediated GLUT1 S-palmitoylation promotes glioblastoma glycolysis and tumorigenesis. *Nat. Commun.* 12, 5872. 10.1038/s41467-021-26180-4 34620861 PMC8497546

[DMM050814C185] Zhang, S., Lv, K., Liu, Z., Zhao, R. and Li, F. (2024). Fatty acid metabolism of immune cells: a new target of tumour immunotherapy. *Cell Death Discov.* 10, 39. 10.1038/s41420-024-01807-9 38245525 PMC10799907

[DMM050814C186] Zhao, H., Yang, L., Baddour, J., Achreja, A., Bernard, V., Moss, T., Marini, J. C., Tudawe, T., Seviour, E. G., San Lucas, F. A. et al. (2016). Tumor microenvironment derived exosomes pleiotropically modulate cancer cell metabolism. *Elife* 5, e10250. 10.7554/eLife.10250 26920219 PMC4841778

[DMM050814C187] Zhu, Z., Achreja, A., Meurs, N., Animasahun, O., Owen, S., Mittal, A., Parikh, P., Lo, T.-W., Franco-Barraza, J., Shi, J. et al. (2020). Tumour-reprogrammed stromal BCAT1 fuels branched-chain ketoacid dependency in stromal-rich PDAC tumours. *Nat. Metab* 2, 775-792. 10.1038/s42255-020-0226-5 32694827 PMC7438275

